# Assessment of the impact of CT calibration procedures for proton therapy planning on pediatric treatments

**DOI:** 10.1002/mp.15062

**Published:** 2021-07-20

**Authors:** Esther Bär, Charles‐Antoine Collins‐Fekete, Vasilis Rompokos, Ying Zhang, Mark N. Gaze, Alison Warry, Andrew Poynter, Gary Royle

**Affiliations:** ^1^ Department of Medical Physics and Biomedical Engineering University College London London UK; ^2^ Department of Radiotherapy Physics University College London Hospitals NHS Foundation Trust London UK; ^3^ Department of Oncology University College London Hospitals NHS Foundation Trust London UK

**Keywords:** dual‐energy CT, imaging for protons, paediatric cancer, paediatric treatment planning

## Abstract

**Purpose:**

Relative stopping powers (RSPs) for proton therapy are estimated using single‐energy computed tomography (SECT), calibrated with standardized tissues of the adult male. It is assumed that those tissues are representative of tissues of all age and sex. Female, male, and pediatric tissues differ from one another in density and composition. In this study, we use tabulated pediatric tissues and computational phantoms to investigate the impact of this assumption on pediatric proton therapy. The potential of dual‐energy CT (DECT) to improve the accuracy of these calculations is explored.

**Methods:**

We study 51 human body tissues, categorized into male/female for the age groups newborn, 1‐, 5‐, 10‐, and 15‐year‐old children, and adult, with given compositions and densities. CT numbers are simulated and RSPs are estimated using SECT and DECT methods. Estimated tissue RSPs from each method are compared to theoretical RSPs. The dose and range errors of each approach are evaluated on three computational phantoms (Ewing's sarcoma, salivary sarcoma, and glioma) derived from pediatric proton therapy patients.

**Results:**

With SECT, soft tissues have mean estimation errors and standard deviation up to (1.96 ± 4.18)% observed in newborns, compared to (0.20 ± 1.15)% in adult males. Mean estimation errors for bones are up to (−3.35 ± 4.76)% in pediatrics as opposed to (0.10 ± 0.66)% in adult males. With DECT, mean errors reduce to (0.17 ± 0.13)% and (0.23 ± 0.22)% in newborns (soft tissues/bones). With SECT, dose errors in a Ewing's sarcoma phantom are exceeding 5 Gy (10% of prescribed dose) at the distal end of the treatment field, with volumes of dose errors >5 Gy of $V_{\text{diff}>5}\;=\;4630.7$ mm^3^. Similar observations are made in the head and neck phantoms, with overdoses to healthy tissue exceeding 2 Gy (4%). A systematic Bragg peak shift resulting in either over‐ or underdosage of healthy tissues and target volumes depending on the crossed tissues RSP prediction errors is observed. Water equivalent range errors of single beams are between −1.53 and 5.50 mm (min, max) (Ewing's sarcoma phantom), −0.78 and 3.62 mm (salivary sarcoma phantom), and −0.43 and 1.41 mm (glioma phantom). DECT can reduce dose errors to <1 Gy and range errors to <1 mm.

**Conclusion:**

Single‐energy computed tomography estimates RSPs for pediatric tissues with systematic shifts. DECT improves the accuracy of RSPs and dose distributions in pediatric tissues compared to the SECT calibration curve based on adult male tissues.

## 1 INTRODUCTION

Proton therapy is an advanced treatment modality with advantages over photon radiotherapy because of improved normal tissue sparing.^1^, ^2^ This is particularly valuable in pediatric practice. Children treated with radiotherapy require special care due to their higher sensitivity to radiation‐induced cancer, higher likelihood of genetic predisposition, and the larger effect of scattered radiation.^3^ The main advantage in proton therapy for children is the reduced low‐dose bath, potentially resulting in lower radiation‐induced toxicity rates. Proton therapy has been shown in previous studies to successfully reduce the dose to normal tissues and achieve outcomes comparable or improved to IMRT in pediatric patients.^4^, ^5^, ^6^, ^7^, ^8^ St. Claire et al. reported a substantial normal‐tissue sparing with protons compared to intensity‐modulated radiotherapy in a pediatric medulloblastoma patient.^9^ Mirabell et al. concluded that protons reduce the risk of secondary cancer by a factor larger than 2.^10^ Comparative studies presenting follow‐up data of brain cancer patients treated with protons versus photons suggest that proton therapy might have a clinical advantage in terms of preserving IQ.^11^, ^12^, ^13^

Despite the clear dosimetric advantages and high degree of normal tissue sparing, there is an increased incidence of unexpected toxicities, especially in younger children. Indelicato et al. investigated brain stem toxicities in 313 pediatric patients following proton therapy, showing that patients younger than 5 years had a much‐increased brain stem toxicity rate than patients older than 5 (6.9% vs. 1.1%).^14^ Further pediatric cases with observed unexpected toxicities are presented by Gunther et al. and Peeler et al.^15^, ^16^

In current clinical practice, dose calculations of pediatric treatment plans are performed using the same approximations as for adult patients. Proton therapy planning and dose calculation is most commonly based on a single‐energy CT (SECT) image of the patient. In a procedure called stoichiometric calibration,^17^ the relative stopping power (RSP) values necessary for treatment planning and dose calculation are estimated voxelwise from CT numbers. The procedure is using a calibration curve constructed from calculated CT numbers and RSPs of standardized adult male human tissues.^18^, ^19^ In practice, centers can use a smaller electron density phantom to characterize the CT spectrum for small patients to account for beam hardening conditions being different in these cases. To generate the calibration curve, however, the compositions and densities of standard adult tissues are used.

The ICRU report 46^20^ acknowledges that “Rapid variations in the components of soft tissues occur in the first 6 months of life, after which more gradual changes take place. Both soft tissues and skeletal tissues exhibit reductions in water content with increasing age.” According to composition studies presented by White et al.,^21^ the compositions and densities of some pediatric tissues can be substantially different from adults. They report, for example, that the carbon content in newborns can be as low as 20.5%, as compared to 68.1% in adults. Conversely, the oxygen content in newborns can be as high as 76.2%, as compared to 19.8% in adults.

This paper focuses on the elemental compositions and densities of pediatric tissues and how well they are described when using a SECT stoichiometric calibration curve for RSP estimation. For adults, a full list of reference compositions and densities exists for radiotherapy‐relevant organs and tissues. The ICRP publication 110^22^ lists 53 different tissues for the standard male and female to calculate organ dose coefficients for internal and external radiation, including 20 bone tissues, 29 soft tissues, teeth, urine, the content of the GI tract, and air inside the body. For pediatric tissues, the availability of elemental compositions and densities is limited. White et al.^21^ list elemental compositions and densities for 5 different age groups and 12 different tissue types. The age groups include fetus (8–40 weeks from conception), newborn (fully grown 40‐week fetus), infant (0 to 18 months), child (1 to 18 years), and young adult (approximately 18 years). The composition data from White et al. were adapted into the ICRU report 46,^20^ which recommends that age dependencies should be taken into account when dosimetric data are calculated. In an effort to develop pediatric voxelized phantoms^23^, ^24^ for radiation dosimetry and radiation protection, standardized elemental compositions and densities of 53 body tissues were derived for pediatric patients by the University of Florida (UF),^25^, ^26^, ^27^ which have now been published in ICRP Publication 143.^28^

While age dependencies in body tissue density and composition are already taken into account in areas like radiation protection, the dose in pediatric proton therapy planning is calculated using standardized adult tissues. This paper investigates the impact on the accuracy of estimated pediatric tissue RSPs for proton therapy planning and dose calculation. Potential treatment inaccuracies resulting from ignoring the differences in body tissues between adult and pediatric patients are explored. Dual‐energy CT (DECT) is evaluated as an error mitigation method.

## 2 MATERIALS AND METHODS

### 2.1 Body compositions

The elemental compositions and densities of pediatric tissues used in this work are taken from the UF hybrid phantom family.^23^, ^24^ The family consists of computational, voxelized phantoms at the reference ages defined in the ICRP Publication 89: newborn, 1‐year‐old, 5‐year‐old, 10‐year old, 15‐year‐old, and adult. For all ages, tissue compositions and densities are given for male and female patients. In the age groups from newborn to 10‐year old, most tissues are assumed to have the same compositions in male and female patients, differentiation is made in ovaries/testes, uterus/prostate, and breast glandular tissue.

The tissue elemental compositions for the phantom family were derived by Wayson,^26^ using the available data on tissue compositions of pediatric patients from the ICRU report 46^20^ and ICRP Publication 89.^29^ Tissue compositions were weighted with the average blood volume per organ^25^ to obtain a tissue mixture that represents the tissues as seen in a voxelized CT scan, using equations (1) and (2) of the ICRP Publication 110. For the same reason, skeletal tissues were re‐segmented following Zankl et al. and Pafundi et al.^27^, ^30^ The constituents of bones are cortical bone, trabecular bone, red bone marrow, yellow bone marrow, cartilage, and endosteum. However, the dimensions of the trabecular cavities containing marrow and endosteum are smaller than the resolution of a CT scan and will hence result in a volume‐averaged CT number. In an attempt to represent the spatial distribution of these tissues, Zankl et al. divide the skeleton into 19 different bones and use the red bone marrow versus cortical bone ratios for each individual bone as given in ICRP Publication 70.^31^ The compositions of the resegmented tissues are calculated as mixtures of the constituents. Furthermore, age‐dependent volumes and masses of the different skeletal tissues are considered.^27^ A list of the tissues considered in this work can be found in Appendix C of this work, a full list of elemental compositions and densities can be found in ICRP Publication 143,^28^ Annex B.

### 2.2 Calculation of theoretical CT numbers and RSP values

In order to investigate if pediatric tissues are represented by the SECT stoichiometric calibration curve, we use the given elemental compositions and densities of each tissue at each age to theoretically calculate their CT number. Theoretical CT numbers of the investigated body tissues are calculated using generic CT spectra with peak voltages of 80, 100, 120, and 140 kVp to cover the range of spectra used for radiotherapy planning.^32^ While the spectra for 80, 100, and 120 kVp were calculated using the *SpekCalc* software,^33^ the spectrum of 140 kVp resembles the unfiltered 140‐kVp spectrum of a Siemens Somatom Definition Flash scanner (Siemens Healthineers, Erlangen, Germany). See Appendix A for an illustration of the used spectra. For comparability with our DECT investigations, we perform all analysis presented in this paper with the 140‐kVp spectrum. Results from the simulated spectra are given in the Appendix to this paper. For all 13 elements abundant in human tissues, the elemental mass attenuation coefficient is taken from the XCOM database,^34^ averaged over the spectrum and weighted according to the elemental compositions in each tissue.

Similarly, the theoretical CT numbers of an electron density phantom Gammex RMI 467 (Gammex Inc, Middleton, WI, USA) are calculated for the energy calibration in the SECT stoichiometric calibration. The elemental compositions and densities from the Gammex RMI 467 phantom are taken from Bourque et al.^35^

Theoretical RSP values of the investigated body tissues and the RMI phantom are calculated using the Bethe formula^36^ using elemental *I*‐values from Bär et al.,^37^ for a proton beam of 100 MeV. Henceforth, these will be referred to as RSP_ref_.

### 2.3 CT calibration

To investigate RSP errors arising from CT calibration methods in pediatric patients, we apply three different approaches to estimate pediatric and adult tissue RSP from the simulated CT numbers. The first approach employs the SECT stoichiometric calibration by Schneider et al.^17^ using a reference dataset of adult tissues to adjust the calibration curve for the use in human tissues. The second approach is a child‐specific SECT calibration. In this approach, we use the Schneider calibration method but the reference data set of adult tissues is replaced by the pediatric tissues described in Section 2.1. The third approach uses dual‐energy CT to estimate the RSP values from CT numbers. Our implementations of each method are detailed below.

#### 2.3.1 Conventional single‐energy CT

**FIGURE 1:**
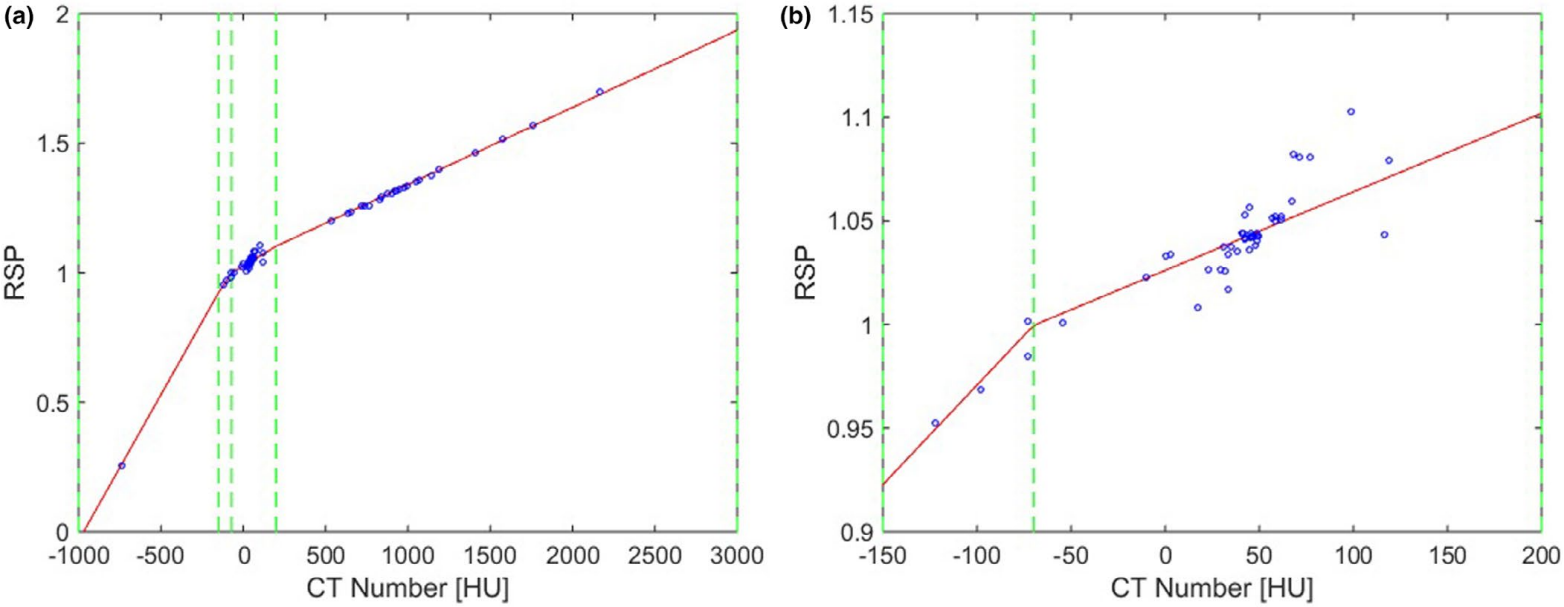
The calibration curve derived for the stoichiometric calibration. The points correspond to the 70 reference tissues, the solid line is the piece‐wise fit and the dashed line indicate the fitting regions. (a) Calibration curve for all reference tissues; (b) region between −150 and 200 HU [Color figure can be viewed at wileyonlinelibrary.com]

The stoichiometric calibration proposed by Schneider et al.^17^ is used in this paper to estimate RSP values from CT numbers. The theoretically calculated CT numbers from the Gammex RMI 467 are used to find the fit parameters $K^{\text{ph}}$, $K^{\text{coh}}$, and $K^{\text{KN}}$ from eq. 8 in Schneider et al. Those parameters are then used to calculate the CT numbers of 70 reference adult tissues, taken from Woodard and White^19^ and White et al.^18^ To generate the calibration curve, we piece‐wise fit the calculated CT numbers to theoretically calculated RSP values for the reference tissues, using the same energy and I‐values as in Section 2.2. We use the 70 tissues listed in Woodard and White as calibration tissues instead of the 53 tissues described in Section 2.1 to have different calibration and testing tissues. Between the different databases, the only duplicate tissues are blood and cartilage. The first linear curve fits lung (−1000 to −150 HU), the second fits adipose tissue (−150 to −85/‐75/‐75/‐95 HU for 80, 100, 120, and 140 kVp, respectively), the third fits soft tissues including thyroid and cartilage (−85/‐75/‐75/‐95 to 200 HU), and the fourth fits bones (200 to 3100 HU). The obtained calibration curve can be found in Figure 1. The theoretically calculated CT numbers for pediatric tissues (Section 2.2) are applied to the calibration curve to find the model estimated RSP values (RSP_SECT_). The model estimated RSP values are compared to the theoretically calculated RSP values (RSP_ref_). The RSP error between estimated and theoretical values is calculated as follows:

(1) $$\Delta \text{RSP}=\frac{{\text{RSP}}_{\text{SECT}}‐{\text{RSP}}_{\text{ref}}}{{\text{RSP}}_{\text{ref}}}.$$

#### 2.3.2 Age‐specific single‐energy CT

**FIGURE 2:**
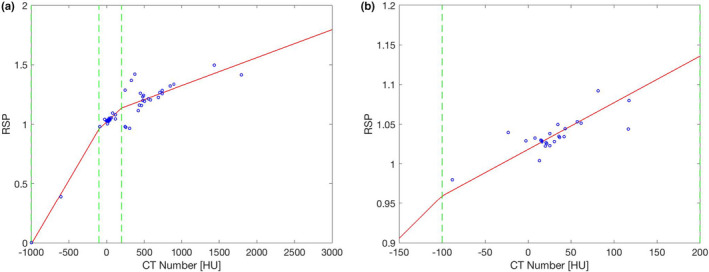
An exemplary age‐specific calibration curve for the 1‐year old female. The points correspond to the 53 calibration tissues of the 1‐year old female, the solid line is the piece‐wise fit and the dashed line indicates the fitting regions. (a) Calibration curve for all reference tissues; (b) region between −150 and 200 HU [Color figure can be viewed at wileyonlinelibrary.com]

To derive the age‐specific single‐energy CT calibration, we use the fit parameters $K^{\text{ph}}$, $K^{\text{coh}}$, and $K^{\text{KN}}$ from above (140‐kVp spectrum) to calculate the CT numbers of the 53 pediatric tissues, taken from ICRP Publication 143^28^ and described above (Section 2.1). Linear curves are fitted section‐wise using the so calculated CT numbers and theoretical RSP values per age group and sex. The fit sections are lung (−1000 to −100 HU), soft tissue (−100 to 200 HU), and bones (200 to 4100 HU). In contrast to the calibration curve obtained with the 70 reference tissues in Woodard and White, the tissues in ICRP publication 143 only contain one adipose tissue so we include it into the soft tissue region. With this procedure, we obtain 12 different calibration curves for 6 different age groups, male and female. An exemplary age‐specific calibration curve for the 1‐year‐old female is shown in Figure 2. The simulated CT numbers for pediatric tissues from Section 2.2 (140‐kVp spectrum) are applied to the calibration curve corresponding to the respective age group and sex to find the model‐estimated RSP values. The model‐estimated RSP values are compared to the theoretically calculated RSP values using Equation (1). It should be noted that the RSP errors obtained this way are self‐consistency errors because the tissues used for calibration are equal to the tissues used for evaluation, since only one set of pediatric tissues is available. This is in contrast to the method above, where we use two different sets of reference tissues for calibration and evaluation.

#### 2.3.3 Dual‐energy CT

To investigate if DECT can reduce the error in RSP estimation for pediatric tissues, the DECT stoichiometric calibration by Bourque et al.^35^ is implemented. Theoretical CT numbers are calculated using generic CT spectra with 80 and 140 kVp with tin filtration to achieve the best possible spectral separation (see Appendix A for spectra). The spectra resemble those of a Siemens Somatom Definition Flash dual‐source dual‐energy scanner. As described in Section 2.2, the CT numbers are calculated for the investigated pediatric tissues as well as for the electron density phantom Gammex RMI 467. The calibration procedure follows the one described in Section 7 of Bourque et al., using theoretically calculated CT numbers instead of a CT scan.

The CT numbers of the pediatric tissues described in Section 2.1 are used to estimate their effective atomic numbers and electron densities following equations (35)–(37) in Bourque et al., but using the elemental *I*‐values from Bär et al.^37^ The *I*‐values of the tissues are estimated by parameterizing the *I*‐value as a function of the effective atomic number, following Equation (18) in Bourque et al. From the estimated electron densities and *I*‐values, an estimate of the RSP (here referred to as RSP_DECT_) is calculated using the Bethe equation.

### 2.4 Patient data sets and construction of virtual phantoms

To evaluate the influence of the RSP errors inferred to pediatric tissues by the SECT stoichiometric calibration on pediatric proton therapy, we use three patient cases. The patients were chosen to represent typical pediatric proton therapy patients with different age and treatment site. The three cases are:

1. An 11.5‐year‐old male patient with a pelvic Ewing's sarcoma;
2. An 18‐year‐old male with a salivary sarcoma;
3. A 5‐year‐old male with a glioma.

For each patient, target volumes, organs relevant for treatment planning and bone structures are contoured by a radiation oncologist using a clinical Varian Eclipse (Varian Medical Systems, Inc., Palo Alto, CA, USA) treatment planning system (TPS).

The patient images are used to create virtual phantoms by assigning a tissue with known elemental composition and density to each voxel, as explained in detail in Remy et al.^38^ The tissue assignment scheme is guided by the clinician‐assigned contours following the AAPM TG‐186 report^39^ on model‐based dose calculation methods. For each contour, a selection of tissues is made (see tissue assignment in Appendix B) from the list of pediatric tissues (Appendix C) based on the expected tissues within the respective contour. After the tissue assignment, a k‐means clustering algorithm (SKLearn, Python^40^) is run on the contours’ CT number probability density function to partition optimally the tissues within the contours into the pre‐assigned tissues. Densities are assigned by sampling a normal distribution centered on the tissue mean density with a 3‐sigma variation of 3σ = 1.7% as characterized by Yang et al.^41^ This way, all voxels within a segmented tissue have the same elemental composition, but a density distribution rather than a single density.

The assigned compositions and densities per CT voxel are used to calculate theoretical CT numbers as described in Section 2.2, using the 140‐kVp CT spectrum and the mass attenuation coefficients from the XCOM database. Gaussian noise with a standard deviation of 7 HU was added to the images. The CT numbers are then used to estimate the RSP voxelwise via the conventional, non‐age‐specific SECT stoichiometric calibration curve (Figure 1).

Similarly, theoretical CT numbers for the three patients are calculated voxelwise for the two DECT spectra to generate virtual DECT images. Gaussian noise of 12 HU (for the 80‐kVp image) and 8 HU (for the 140‐kVp image) is added to the images. The noise was selected to be equivalent to the noise in the SECT image in terms of dose to water, as described in Bär et al.^42^ The DECT images of the three phantoms are used to voxelwise estimate the RSP values (RSP_DECT_).

We hence obtain three maps of RSP values for each of the three patients: (1) The theoretically calculated reference RSP (RSP_ref_); (2) the RSP estimated from the stoichiometric calibration (RSP_SECT_); and (3) the RSP estimated from the DECT calibration (RSP_DECT_).

### 2.5 Treatment plan evaluation

For the three phantoms, the RSP maps (reference, SECT and DECT) are imported into the clinical TPS Eclipse (Varian, Medical Systems, Inc., Palo Alto, USA). One treatment plan per phantom is generated based on the RSP_SECT_ maps, as it would be done during clinical treatment planning. All plans are designed as a spot scanning proton treatment and were optimized using conventional (i.e., non‐robust) optimization. For the first phantom (based on the Ewing's sarcoma patient), two fields at gantry angles 15° and 170° are used. The plan was optimized using multifield optimization intensity modulated proton therapy (IMPT). A total dose of 50.4 Gy is prescribed to the high‐dose CTV, a dose of 45 Gy is prescribed to the low‐dose CTV. For the second phantom (based on the salivary sarcoma patient), two fields at gantry angles 270° and 330° and single‐field optimization IMPT are used. The prescription dose is 64.8 Gy to the CTV. For the third phantom (glioma patient), two fields at gantry angles 290° and 235° and single‐field optimization IMPT are used. The prescription dose is 54 Gy.

For plan evaluation, a robustness analysis is performed by introducing uncertainty parameters to the isocenter position and the calibration curve. For the Ewing's sarcoma phantom, an uncertainty on the isocenter position of 5 mm and 3.5% on the calibration curve are assumed. For the salivary sarcoma and glioma, we use 3 mm and 3.5% uncertainties, as commonly assumed in head treatments with mask fixation. For the robustness analysis, the treatment plan is recalculated 12 times taking into account the isocenter shift in either direction or dimension (positive and negative shift along the X‐, Y‐, and Z‐axes) as well as adding and subtracting the calibration curve uncertainty. The plan was deemed acceptable if 99% of the prescription volume received at least 95% of the prescribed dose for all 13 plan scenarios. Additional acceptance criteria were dose uniformity and the tolerance of the surrounding organs.

The plan is then used to recalculate the dose on the RSP_ref_ map containing the ground truth RSP values, to represent the dose that would be delivered to those tissues. The dose differences between the planned dose (based on the SECT‐estimated RSP map) and the simulated delivered dose (based on the theoretically calculated RSP map) are evaluated. Furthermore, the plan is used to recalculate the dose on the RSP_DECT_ map for comparison to SECT‐estimated and simulated delivered dose.

In addition to the dose differences, we evaluate the difference in beam range and water equivalent range (WER) between treatment beams calculated on the different RSP maps. The beam range is calculated for each beam angle by taking profiles of the dose distribution along the beam's path. A profile is taken along all those lines in the dose file with a dose exceeding 20% of the prescribed dose. For each profile, the position of the 80% dose fall‐of $R_{80}$ is found via interpolation. The mean absolute differences ($\Delta R_{\text{MAE}}$) and standard deviations between the dose calculated on RSP_ref_ versus RSP_SECT_ and RSP_DECT_ are determined. To assess the WER for the three phantoms and each treatment beam, we use the 5 most distal energy layers and an in‐house written ray‐tracing algorithm. Ray tracing is performed from the skin to the Bragg peak, considering the energy of each spot. We compare the mean absolute WER difference between the WER calculated on the theoretical images (WER_ref_) to those calculated on SECT (WER_SECT_) and DECT (WER_DECT_) images.

## 3 RESULTS

### 3.1 RSP estimation errors in pediatric tissues

Theoretically calculated CT numbers and RSPs, SECT‐estimated RSPs (140‐kVp spectrum) as well as RSP errors for all tissues at all ages are listed in Appendix C, the corresponding DECT data are listed in Appendix D. Statistics of the RSP estimation errors for all pediatric tissues as well as adult tissues are presented in Table 1. We group the tissues into soft tissues (tissues 22 to 52 in the tables in Appendix C and D) and bones (tissues 2 to 21). Results exclude teeth and air inside the body. The table shows the mean error (μ), standard deviation (σ), and minimum and maximum error per age and sex for soft tissues and bones separately. For consistency with DECT, we show here the SECT results using the 140‐kVp spectrum but have listed the corresponding results from the simulated spectra of other tube voltages in Appendix E. For SECT (upper part of Table 1), it is observed that the RSP for pediatric soft tissues are systematically overestimated using the stoichiometric calibration, with mean errors ranging from 1.35% to 1.96% as opposed to 0.19% in adult female soft tissues. It should be noted that certain pediatric soft tissues with large RSP errors, such as the tissue within the medullary cavity of the long bones (especially tissues number 22, 23, and 25 in Appendix C) are scarce within the human body, and only used in this study in the Ewing's sarcoma phantom. While influencing the mean and standard deviation RSP errors over all tissues, their contribution to the range errors presented in this study are small.

**TABLE 1:** Percentage mean relative stopping power (RSP) errors, standard deviations, and minimum and maximum errors for all age groups (female and male) estimated with conventional single‐energy computed tomography (SECT) (upper part of the table), age‐specific SECT (middle part of the table), and dual‐energy computed tomography (DECT) (lower part). Teeth and air inside the body are excluded from the statistics

Age groups	Soft tissues				Bones			
	μ	σ	min	max	μ	σ	min	max
SECT 140kVp								
Newborn female	1.96	4.18	−2.79	14.58	−3.35	4.76	−13.97	2.31
1‐year‐old female	1.86	4.82	−3.14	17.80	−2.99	6.27	−18.65	5.13
5‐year‐old female	1.40	3.46	−3.13	14.05	−2.64	4.88	−13.55	3.58
10‐year‐old female	1.30	3.03	−3.13	10.40	−1.57	4.31	−11.49	4.83
15‐year‐old female	1.35	3.17	−3.14	12.65	−1.16	4.15	−9.77	6.10
Adult female	0.19	1.13	−2.60	3.93	0.54	2.55	−4.17	5.42
Newborn male	1.96	4.18	−2.79	14.58	−3.35	4.76	−13.97	2.31
1‐year‐old male	1.90	4.80	−3.14	17.80	−2.99	6.27	−18.65	5.13
5‐year‐old male	1.44	3.44	−3.13	14.05	−2.64	4.88	−13.55	3.58
10‐year‐old male	1.34	3.01	−3.13	10.40	−1.57	4.31	−11.49	4.83
15‐year‐old male	1.38	3.21	−3.13	12.84	−1.16	4.20	−9.86	6.24
Adult male	0.20	1.15	−2.60	3.93	0.10	0.66	−2.35	0.58
Age‐specific SECT								
Newborn female	1.68	5.23	−1.96	17.65	−2.26	4.44	−11.61	3.34
1‐year‐old female	1.95	5.69	−3.36	20.24	−2.31	5.97	−17.07	6.72
5‐year‐old female	1.24	4.33	−3.40	17.05	−1.59	4.61	−11.16	5.14
10‐year‐old female	1.07	3.25	−3.28	10.49	−1.03	4.45	−11.28	5.12
15‐year‐old female	1.25	3.27	−3.66	11.53	−1.25	4.38	−10.25	6.06
Adult female	0.08	1.68	−2.98	7.72	0.23	2.44	−4.65	4.77
Newborn male	1.68	5.23	−1.96	17.65	−2.26	4.44	−11.61	3.34
1‐year‐old male	1.95	5.69	−3.68	20.23	−2.32	5.97	−17.08	6.71
5‐year‐old male	1.24	4.32	−3.72	17.04	−1.59	4.61	−11.17	5.13
10‐year‐old male	1.07	3.24	−3.30	10.47	−1.04	4.45	−11.29	5.12
15‐year‐old male	1.27	3.30	−3.64	11.67	−1.27	4.42	−10.34	6.03
Adult male	0.17	1.66	−2.83	7.67	0.02	0.21	−0.28	0.49
DECT								
Newborn female	0.17	0.13	−0.23	0.38	0.23	0.22	−0.23	0.61
1‐year‐old female	0.07	0.15	−0.22	0.38	0.15	0.23	−0.25	0.71
5‐year‐old female	0.02	0.19	−0.61	0.38	−0.07	0.26	−0.45	0.76
10‐year‐old female	−0.01	0.28	−0.92	0.38	−0.37	0.41	−0.97	0.73
15‐year‐old female	−0.03	0.34	−1.36	0.38	−0.51	0.50	−1.15	0.72
Adult female	0.10	0.12	−0.10	0.38	−0.74	0.45	−1.26	0.52
Newborn male	0.17	0.13	−0.23	0.38	0.23	0.22	−0.23	0.61
1‐year‐old male	0.08	0.14	−0.22	0.38	0.15	0.23	−0.25	0.71
5‐year‐old male	0.03	0.19	−0.61	0.38	−0.07	0.26	−0.45	0.76
10‐year‐old male	−0.01	0.28	−0.92	0.38	−0.37	0.41	−0.97	0.73
15‐year‐old male	−0.02	0.34	−1.37	0.38	−0.53	0.51	−1.16	0.72
Adult male	0.10	0.12	−0.08	0.38	−0.63	0.44	−1.02	0.52

Bone RSPs in pediatric tissues are systematically underestimated, with mean errors ranging from −3.35% to −1.16% as opposed to 0.54% in the adult female and 0.10% in the adult male. For pediatric tissues, estimation errors are between −18.65%, observed in the spongiosa of the ulnae and radii bones of the 1‐year‐old male and female, and 17.80%, observed in the medullary cavity of the humeri and femora bones. For male adult tissues, the errors are between −2.60% in skin tissue and 3.93% in lung tissue. For female adult tissues, however, errors are between −4.17% in the spongiosa of the cranium and 5.42% in the spongiosa of the scapulae. The results obtained using simulated CT spectra with different tube potentials are very similar to those reported for the 140‐kVp spectrum (Appendix E). For example, using the 80‐kVp spectrum we find mean errors as high as 2.13 ± 5.19% (soft tissues) and −3.53 ± 6.91% (bones) in the 1‐year‐old female, comparable to the results shown in Table 1 from the 140‐kVp spectrum. The middle part of Table 1 presents the RSP estimation errors achieved with age‐specific SECT calibration curves. It is observed that for all age groups, the mean estimation errors for soft tissues and bones are reduced compared to the SECT curve derived with adult tissues, whereas standard deviations, minimum, and maximum prediction errors are still high, especially for pediatric age groups. Statistics of the RSP estimation error using DECT are presented in Table 1, lower part. We observe that DECT reduces the RSP estimation errors in pediatric tissues as compared to SECT. In soft tissues, low mean errors are observed, ranging from −0.03% in 15‐year‐olds to 0.17% in newborns. Standard deviations in soft tissues are below 0.34% (observed in the 15‐year‐old female/male). Minimum and maximum errors are well within 1%, with one exception (minimum error in the 15‐year‐old female/male, observed in the medullary cavity of the humeri and femora bones). A similar observation is made in the bone tissues, where the mean errors in pediatric tissues range from −0.53% to 0.23%, with standard deviations of 0.51% or less.

**FIGURE 3:**
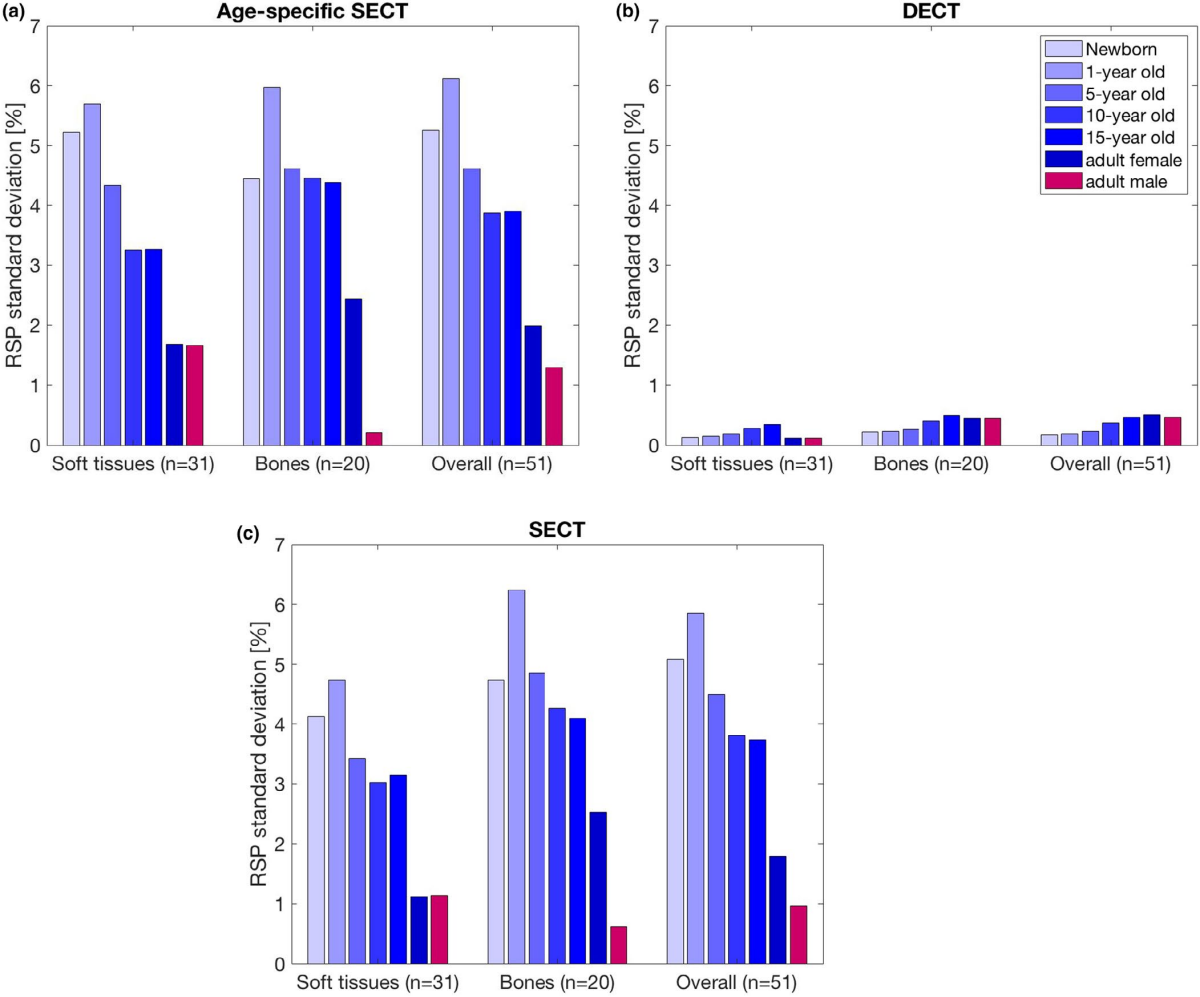
The standard deviation of the Relative stopping powers estimation error for soft tissues, bones and over all tissues for the different age groups. The left panel (a) shows the standard deviation of the single‐energy computed tomography (CT) estimation using the conventional stoichiometric calibration with adult reference tissues. The right panel (b) shows the standard deviation of the single‐energy CT estimation using age‐specific calibrations. The lower panel (c) shows the standard deviation of the dual‐energy CT estimation. The standard deviation for the male and female newborn, 1‐year old, 5‐year old, and 10‐year old are equal; standard deviations of the 15‐year‐old male and female are very similar (3.76% vs. 3.80%), hence only the female data are shown here [Color figure can be viewed at wileyonlinelibrary.com]

Figure 3a visualizes the standard deviation of the SECT‐based RSP estimation error for soft tissues, bones, and overall. High errors and a large variation are observed in pediatric tissues. Standard deviations of the estimation errors decrease towards adulthood. However, we still find elevated standard deviations in the adult female, with the lowest deviations observed in the adult male. A similar pattern is observed for the age‐specific SECT calibration, displayed in Figure 3b. Figure 3c visualizes standard deviations of the RSP estimation errors with DECT.

**FIGURE 4:**
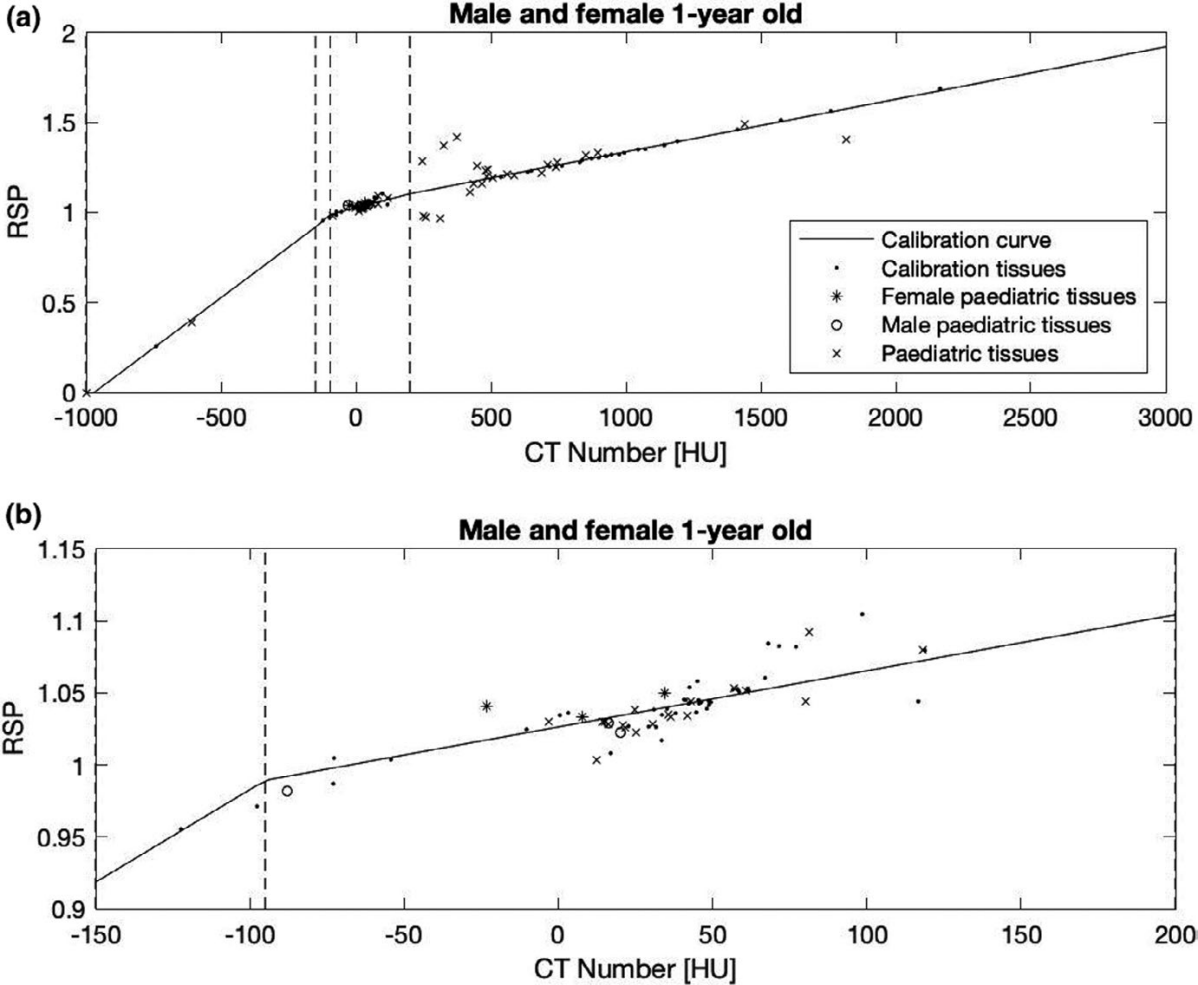
(a) Positions of tissues from a 1‐year‐old child relative to the stoichiometric calibration curve with zoom into the soft tissue region (b). The dots represent the calibration tissues (adult tissues), the line is the piece‐wise fit (Schneider et al. 1996). Tissues that differ in male and female children are marked with a circle (male) or an asterisk (female). In soft tissues, the largest deviation is observed in the medullary cavity of the humerus (17.80%). Please note that this tissue has theoretical computed tomography number of 309 HU and therefore falls within the bone fit. In bones, the highest deviation is observed in the spongiosa tissue of the radius and ulna bones (−18.65%). Similar graphs for the remaining age groups can be found in Appendix F

Figure 4 shows the tissues of a 1‐year‐old child in relation to the SECT calibration curves. The figures for the remaining age groups can be found in Appendix F. The subfigures show the tissues of a 1‐year‐old male/female across (a) the whole CT range and (b) the soft tissue region (−150 to 200 HU). For this age group, most tissues have similar compositions and densities between male and female children. Differences only appear in gender‐specific tissues, such as tissue number 42 in Appendix C (testes vs ovaries), tissue number 46 (prostate vs uterus and cervix), as well as in tissue number 48 (breast glandular tissue). Hence, male and female pediatric tissues are shown in the same graphs, with male tissues marked with a circle and female tissues marked with an asterisk. It should be noted that the soft tissues with the highest RSP errors (tissues 22, 23, and 25) are located within the bone fit and not seen on Figure 4b. It can be seen, however, that most pediatric tissues in the soft tissue region are located below the calibration curve, leading to a systematic overestimation of RSP values.

### 3.2 Treatment plan evaluation

**FIGURE 5:**
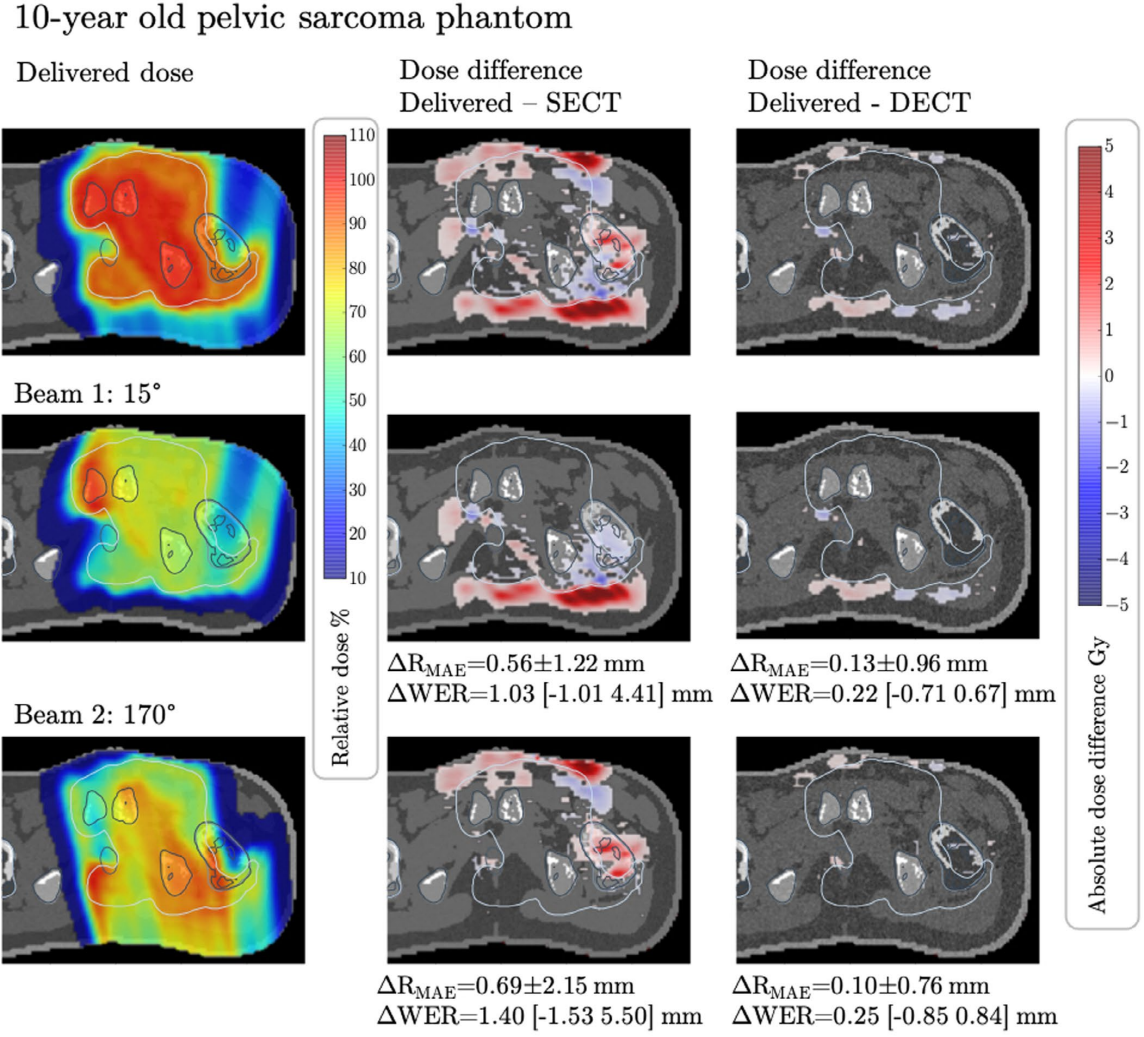
Dose difference between the dose calculated on theoretically calculated Relative stopping powers (RSP) and computed tomography (CT)‐estimated RSP for the Ewing's sarcoma phantom with assigned tissues from a 10‐year‐old male. The upper panels show the total dose distribution and total dose differences between the dose calculated on theoretical RSP maps of the phantom and dose calculated on single‐energy CT‐ and dual‐energy CT‐estimated RPS maps. The middle and lower panels show the dose difference for each treatment beam, with their respective mean absolute range differences (ΔR_MAE_) and mean absolute WER differences. The range of WER differences (min max) is shown in brackets. The greyscale window of the underlying RSP maps is 0.87 to 1.27. Dose differences <0.1 Gy are masked out. The displayed target contour (white) is the PTV [Color figure can be viewed at wileyonlinelibrary.com]

**FIGURE 6:**
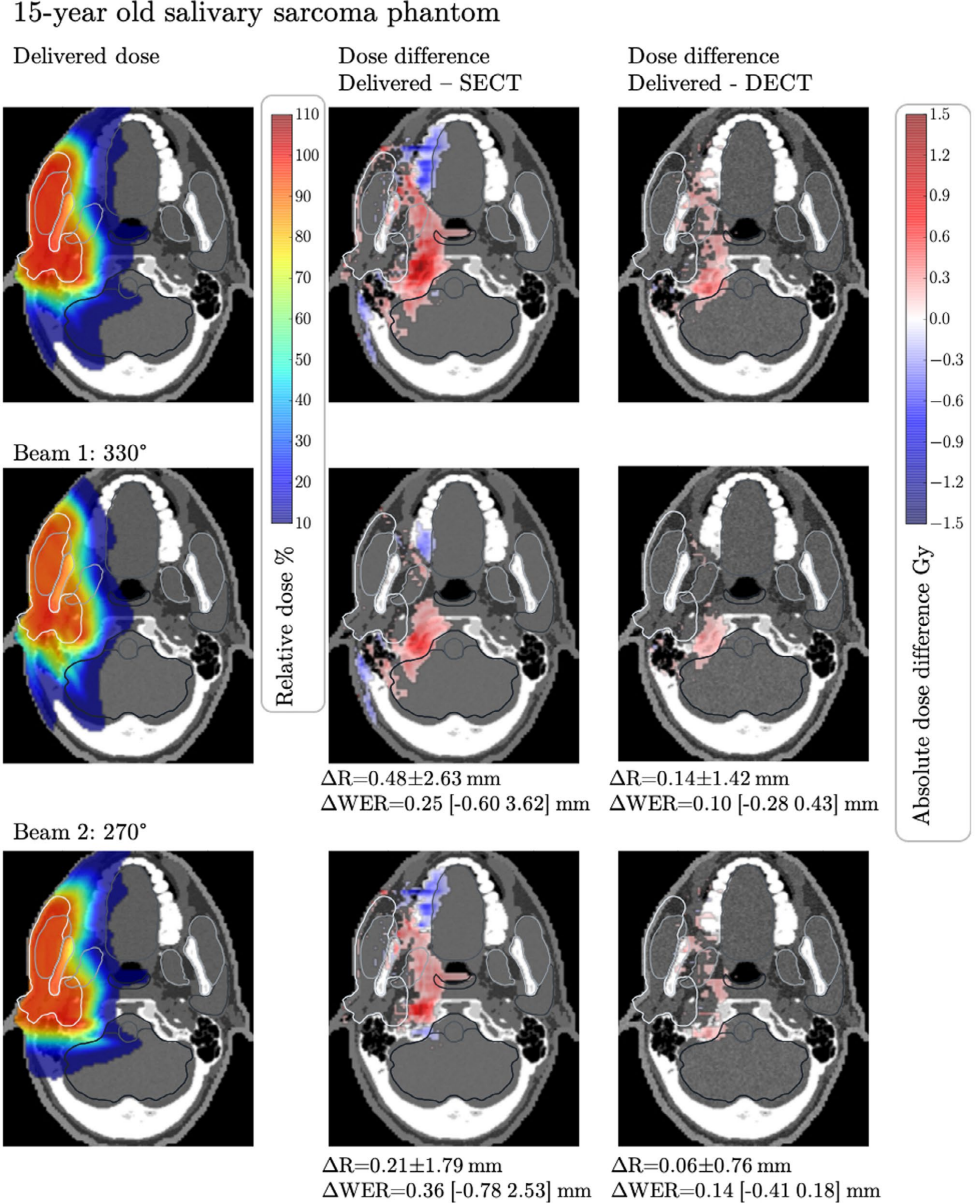
Dose difference between the dose calculated on theoretically calculated Relative stopping powers (RSP) and computed tomography (CT)‐estimated RSP for the salivary sarcoma phantom with assigned tissues from a 15‐year‐old male. The upper panels show the total dose distribution and total dose differences between the dose calculated on theoretical RSP maps of the phantom and dose calculated on single‐energy CT‐ and dual‐energy CT‐estimated RPS maps. The middle and lower panels show the dose difference for each treatment beam, with their respective mean absolute range differences (ΔR_MAE_) and mean absolute WER differences. The range of WER differences (min max) is shown in brackets. The greyscale window of the underlying RSP maps is 0.87 to 1.27. Dose differences <0.1 Gy are masked out. The displayed target contour (white) is the PTV [Color figure can be viewed at wileyonlinelibrary.com]

**FIGURE 7:**
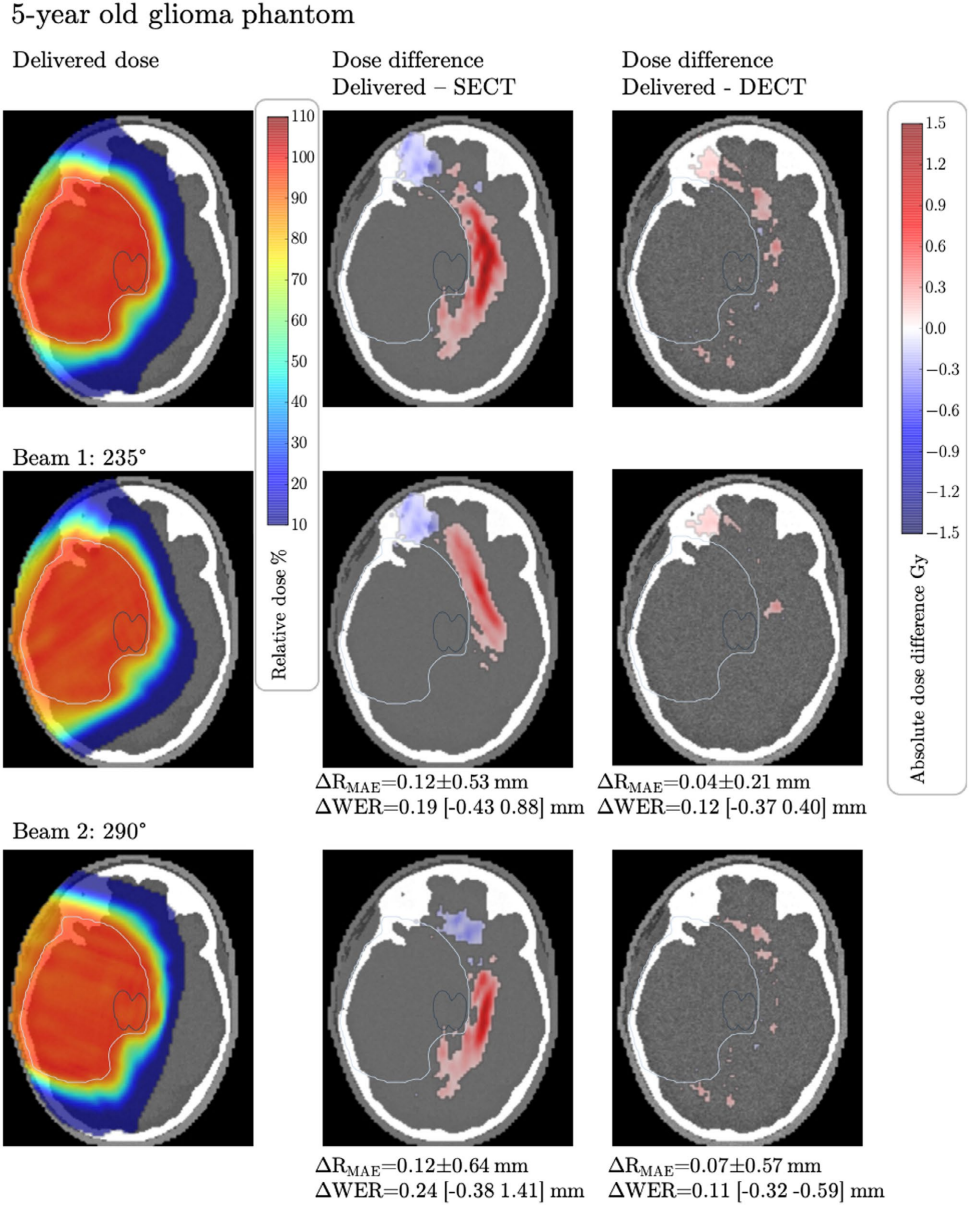
Dose difference between the dose calculated on theoretically calculated Relative stopping powers (RSP) and computed tomography (CT)‐estimated RSP for the glioma phantom with assigned tissues from a 5‐year‐old male. The upper panels show the total dose distribution and total dose differences between the dose calculated on theoretical RSP maps of the phantom and dose calculated on single‐energy CT‐ and dual‐energy CT‐estimated RPS maps. The middle and lower panels show the dose difference for each treatment beam, with their respective mean absolute range differences (ΔR_MAE_) and mean absolute WER differences. The range of WER differences (min max) is shown in brackets. The greyscale window of the underlying RSP maps is 0.87 to 1.27. Dose differences <0.1 Gy are masked out. The displayed target contour (white) is the PTV [Color figure can be viewed at wileyonlinelibrary.com]

Figures 5, 6, 7 show the differences in dose maps calculated on the CT‐estimated RSP maps versus the simulated delivered dose calculated on theoretical RSP maps. Red areas show regions where the simulated delivered dose is higher than the CT‐estimated dose and blue areas show regions where the simulated delivered dose is lower than the CT‐estimated dose. In all three phantoms, we observe that the simulated delivered dose is higher than the SECT‐estimated dose in regions distal to the target volume, indicating that the actual beam range is larger than the estimated beam range, leading to overshoots into normal tissues. Additionally, the target DVHs of the three dose distributions per phantom were compared. For each of the three phantoms, we compared the DVH curves of the target volumes and DVH metrics D_min_, D_max_, D_mean_, and D_95_. For all three treatment plans generated, the DVH curves of the target volume are very similar across the three different RSP maps and do not show any relevant differences. In the DVH metrics comparison, we observe that all investigated metrics agree across the different RSP maps within 0.2 Gy, confirming a robust target coverage under the presented RSP uncertainties. The DVH curves as well as the detailed results of the DVH metrics can be found in Appendix G to this study. In the Ewing's sarcoma phantom, Figure 5, large areas with dose differences between the simulated delivered and SECT‐estimated doses of more than 5 Gy are observed, corresponding to 10% of the prescribed dose. The total volume of tissue within the phantom receiving a dose difference >5 Gy is $V_{\text{diff}>5}\;=\;4630.7$ mm^3^. When using DECT to estimate the RSPs and perform the dose calculation, the discrepancy between the simulated delivered and the estimated dose is reduced. In fact, the dose differences between estimated and simulated delivered dose are in most slices not exceeding 1 Gy, with $V_{\text{diff}>5}\;=\;104.4$ mm^3^.

In the salivary sarcoma phantom, shown in Figure 6, we make similar observations as in the Ewing's sarcoma phantom. The observed dose errors are smaller in this case, in the order of 1 Gy (corresponding to 2% of the low dose–volume prescription), due to the shallower depth and smaller sizer of the target region. The total volume of tissue within the phantom receiving a dose difference >1 Gy is $V_{\text{diff}>1}\;=\;1701.4$ mm^3^. In this particular case, we see that the areas of higher dose can reach critical structures such as the brain stem, the pharynx, and the oral cavity, potentially increasing the amount of normal tissue damage and hence the risk of toxicities. The dose differences between DECT‐estimated and simulated delivered dose are much smaller, showing fewer regions with overshoots and a lower dose in normal tissues ($V_{\text{diff}>1}\;=\;41.7$ mm^3^).

In the glioma patient, Figure 7, we observe an overshoot leading to overdoses larger than 1.5 Gy distal to the target volume ($V_{\text{diff}>1}\;=\;3013.6$ mm^3^), indicating that a higher dose than estimated is given to healthy regions of the brain, that could be spared by using DECT for treatment planning ($V_{\text{diff}>1}\;=\;0.0$ mm^3^).

In line with the observed overdoses, we find that the beam ranges differ between SECT‐estimated and theoretical RSP maps, with range differences between simulated delivered range and SECT‐predicted range (delivered range ‐ SECT range) being systematically positive, implying that the delivered beam travels deeper into tissue than calculated using SECT. For all three phantoms and all six beams, the mean absolute range differences and WER differences are given in Figures 5, 6, 7. Largest differences are observed between SECT and theoretically calculated RSP maps in the Ewing's phantom, where the WER differences of single beam spots range from −1.53 to 5.50 mm (170°). For the salivary phantom, we observe WER differences between −0.60 and 3.62 mm (330°). For the glioma phantom, the WER differences are between −0.38 and 1.14 mm (290°). With DECT, the range differences to the theoretically calculated case are kept well within 1 mm for the Ewing's phantom and well within 0.5 mm for the salivary and glioma phantoms. It should be noted here that the range differences are solely due to CT conversion and do not include other sources of range uncertainties such as biology, *I*‐values or setup.

## 4 DISCUSSION

In this paper, we demonstrate the potential dose error in pediatric proton therapy arising from the SECT stoichiometric calibration based on standardized reference tissues. In proton treatment planning, the potential difference in tissue composition and density is currently not taken into account and treatment planning is performed based on the assumption that tissues do not vary among patients of different age or sex. Considering that the dose depositions of charged particles depends not only on the density but also the elemental compositions of the irradiated tissue, a more age‐specific treatment planning approach, such as an age‐specific SECT calibration curve or the use of DECT, would be desirable.

In a theoretical scope, we use pediatric tissue compositions and densities of different age groups to assess the accuracy of clinical CT conversion methods for pediatric proton therapy patients. We observe large differences in CT numbers and RSPs between the different age groups, without a clear trend or correlation between CT number and RSP. SECT‐estimated RSP values from look‐up tables adapted for either adults or children, have high errors for pediatric tissues reaching as high as 18.6% in some tissues. The high estimation error arises from the calibration curve, which is built on the assumption that there exists a high correlation between density and elemental composition. For adult tissues, the electron density increases approximately monotonically with the photon attenuation coefficient (and CT number), so it is acceptable to use a bijection for the CT number to electron density calibration. This is also nearly true for the CT number to RSP calibration. The assumption of monotony, however, is not valid in pediatric tissues, where the difference in oxygen and carbon content significantly changes photon attenuation and proton stopping powers. It should be noted that the pediatric tissue compositions in White et al.,^21^ on which the tissues used here are based, were obtained by measurements similar to adult tissues listed in Woodard and White.^18^, ^19^ It can therefore be assumed that uncertainties, although not quoted in the literature, are similar for both tissues. It is observed that many pediatric tissues as well as female adult tissues do not exhibit the correlatives traits observed in adult male and are therefore located far away from the calibration curve (Figure 4), resulting in high RSP estimation errors. Examples for such tissues are spongionsa of the different bones (tissues 3–21 in Appendix C) which are systematically underestimated and tissues in the medullary cavity (tissues 22–25 in Appendix C) which are systematically overestimated. It appears that the use of a SECT calibration, or look‐up table approach, even if constructed to be age‐specific, leads to inaccuracies in the dose calculation for pediatric tissues.

### 4.1 Dose and range errors originating from a single calibration curve for pediatric treatment planning

In three pediatric computational phantoms, we show the potential consequences of RSP estimation errors on the delivered proton dose. Three cases were chosen to represent different body sites, depths and sizes of target volumes, and different ages of patients. In all three phantoms, we observe that the simulated delivered dose (and associated beam range) is much higher outside the target volume than planned for, especially in regions distal to the Bragg peak. In addition to the positive dose differences, we find a few regions where the simulated delivered dose is lower than the planned dose (blue dose differences in Figures 5, 6, 7). These occur often after the beam has passed through a substantial amount of tissues where RSP values are overestimated by the CT calibration (e.g., teeth in Figure 6). In these tissues, the actual RSP are lower than estimated, leading to a negative dose difference if comparing CT estimated versus simulated delivered dose. The observed dose errors (positive and negative) exceed 5 Gy in the Ewing's sarcoma patient, a substantial amount of dose given to normal tissues. These dose errors would go completely unnoticed in patients and might cause of long‐term toxicities. The consequences of such dose errors can be considerable, especially when treating lesions near critical structures and in the brain.

Large RSP prediction errors are seen especially in pediatric skeletal tissues, with bones being systematically underestimated. For soft tissues, we observe a systematic overestimation of pediatric tissues using a single calibration curve. The largest errors, however, are seen in tissues in the medullary cavity of the long bones. Since these tissues make up a small portion of the soft tissues abundant in a human body, their influence on the overall range uncertainty might be very small. If these tissues are excluded, the mean RSP error and standard deviation of soft tissues is still overestimated. As an example, the mean RSP error of the soft tissues of a newborn excluding the medullary cavity tissues (tissue numbers 22, 23, and 25) is (0.70 ± 1.50)%. This systematic overestimation, also seen in Figure 5b, is causing the systematic range shift in the planned Bragg peak position observed in the three exemplary cases. This range shift cannot be avoided by using different beam angles (since common to all soft tissues). It might, however, be mitigated by using the age‐specific SECT calibration presented in this paper. Here, the soft tissue RSP error excluding medullary cavity tissues is (0.05 ± 1.34)%. The residual range uncertainty when age‐specific calibration curves are used will be investigated in the future. Similarly, if the use of more treatment fields or different beam angles can mitigate the dose and range deviations was not investigated in this study and will be the subject of future work.

The treatment plans presented in this study were evaluated for their robustness against ±3.5% range uncertainties. This number of 3.5% was chosen because it is often used in robust evaluation and was originally, according to Paganetti,^43^ proposed by Goitein^44^ to include CT number uncertainty and RSP conversion uncertainty. This number, although still in use, was revisited in Paganetti's paper to be 4.6% for parametric (non‐Monte Carlo) dose calculation algorithms. Paganetti's estimate includes uncertainties from CT imaging and calibration, CT conversion to RSP, CT grid size, tissue I‐values, and range degradation due to inhomogeneities. In this paper, we use fixed theoretically calculated tissues and a fixed computational phantom, thus removing the uncertainties from CT imaging and calibration, CT grid size, I‐values, and related range degradation. Therefore, our results shown here should be compared to the 0.5% margin coming from CT‐RSP conversion. A margin of 0.5% would not cover the RSP conversion errors shown here. On the other hand, increasing the margins or robustness parameters to include these RSP uncertainties is not a desirable mitigation method for pediatric cases, where secondary dose to normal tissues can lead to severe long‐term side effects.

### 4.2 Clinical considerations

Radiotherapy is a high‐risk modality for the treatment of cancers in children.^45^, ^46^ A balance must be achieved between the risk of it causing severe, occasionally fatal, adverse effects,^47^, ^48^ and the risks to the life and wellbeing of the child if treatment is not successful. The clinical justification for the use of proton beam radiotherapy rather than photon irradiation in children is centered on changing this balance: a significant reduction in treatment related morbidity for an equal prospect of cure is much more common than improving cure probability through dose escalation facilitated by lower normal tissue doses.

Despite the theoretical, and real, advantages of protons in reducing high doses outside the target volume, and in reducing extensive unnecessary low‐dose exposure to healthy normal tissues, proton beam radiotherapy is still associated with risks. Tumors are often situated very close or immediately adjacent to critical normal structures such as the optic chiasm or brainstem. The dose required to control the tumor may be close to or exceed the tolerance of the normal tissue. The doses used often lie on the steep part of the dose morbidity curve, meaning that a small dose increment may result in a profound adverse effect. To minimize the risk, it is essential that proton radiotherapy planning systems are as precise and accurate as possible, both in estimation of the dose which will be delivered, and its anatomical distribution. Small differences in the physical dose delivered may result in greater adverse effects than would be predicted from dose alone for two reasons. First, the change in relative biological effectiveness, particularly at the Bragg peak, may be greater in reality than has been understood to be the case. Second, as the linear quadratic formula demonstrates, late effects are proportional to the square of the dose per fraction. Together these mean that the adverse effect of a small absolute dose increase close to the tolerance dose of an organ at risk may be greater than might be expected from considering the physical dose alone. Put simply, radiation biology has to be considered alongside physical aspects of radiation dosimetry. Errors in range may result in healthy tissues outside but close to the planning target volume, and believed therefore to be safe, being in fact in part within the true high dose volume.

In the treatment of a brain tumor, for example, requiring a prescribed dose of 54 Gy in 30 × 1.8 Gy fractions, situated adjacent to the optic chiasm or brainstem, presents significant risks. The generally accepted tolerance of these organs at risk with conventionally fractionated treatment is 55 Gy. A 2% error in the physical dose estimate will take the prescribed dose beyond the tolerance dose, and any biological magnification of this will increase the risk further. As the margin for error is so limited, it is absolutely essential that dosimetric calculations are based on the most precise and accurate models and algorithms, if unexpected toxicity is to be avoided.

### 4.3 Dual‐energy CT to mitigate dose and range errors in pediatric treatment planning

In this paper, we stress that current clinical CT imaging and calibration practice might not be the most suitable choice to calculate proton therapy dose distributions for pediatric patients. DECT is presented as a more accurate strategy. We show that DECT is able to accurately estimate RSPs of tissues of all ages and sexes with a similar accuracy, diminishing the need of a calibration curve that is specific to a certain patient group. We furthermore show in computational phantoms that the dose calculated on DECT‐estimated RSP maps is very similar to the simulated delivered dose, with only small dose errors never exceeding 1 Gy. Previous studies already show that DECT can estimate dose distributions in adult patients with a higher accuracy than SECT.^49^, ^50^ In their 2019 paper, Wohlfahrt et al.^50^ show in a cohort of 22 pediatric and 80 adult patients that the SECT look‐up table approach is limited in accurately estimating the RSP values of inter‐ and intra‐patient variations. They conclude that dual‐energy CT is advisable for all patients, allowing for a better estimation of RSPs in the presence of patient and tissue variability. Our results support their conclusion and furthermore show that the benefit for pediatric patients is high due to the difference of elemental compositions and the resulting RSP errors for some tissues when using a look‐up table approach. Additionally, our analyses show that constructing an age‐specific look‐up table for children can account for systematic shifts but not for the large spread of tissues around the curve. Creating a single calibration curve, even if it's purely based on pediatric tissues, always results in severe overestimation of some tissues and severe underestimation of others. While for this paper we have presented DECT as a solution to estimate RSPs for pediatric patients more accurately, other solutions exist. Future work could cover the use of personalized look‐up tables using proton radiography^51^ or the use of proton CT as a low‐dose imaging modality.^52^, ^53^, ^54^

### 4.4 Other remarks

We decided to use the rather uncommon spectrum of 140 kVp to establish our SECT calibration curve. While high tube voltages like this are scarcely used for proton therapy planning of children, we have chosen this spectrum over calculated spectra to be consistent with the employed DECT spectra. However, we have shown with simulated spectra of different tube voltages that the conclusions drawn from this spectrum expand to other spectra and are valid for any kind of SECT‐based look‐up table, regardless of the tube voltage. Bearing in mind that this study is purely theoretical, the conclusions drawn should be verified in a clinical scenario. One limitation of the presented study is the accuracy and reliability of tissue data. The data used here are based on the elemental compositions and densities published by White et al.,^18^, ^19^, ^21^ published between 1986 and 1991. The aforementioned papers quote data collected and published in the 1950s. The accuracy of elemental compositions of human tissues and their variations among age and sex is unclear, without standard deviations quoted and variance analysis performed. New techniques to measure compositions of body tissues might have been developed and might provide new insights into how tissues vary among patients of different ages and sexes.

## 5 CONCLUSION

Our study based on computational phantoms indicates that SECT could lead to systematic RSP errors in pediatric patients. The accuracy of current treatment planning strategies using conventional SECT imaging for RSP estimation for treatment planning could be improved substantially for pediatric patients. In this context, the stoichiometric curve, derived on standardized male adult tissues, is limited for patients other than the adult male. DECT has the capacity to accurately estimate RSP values for patients of all sex and age, showing large improvements in RSP and dose accuracy especially in pediatric patients.

## CONFLICT OF INTEREST

No conflict of interest to disclose.

## Supporting information

Supplementary Material
