# Supplementary material for: Assessment of the impact of CT calibration procedures for proton therapy planning on pediatric treatments
Source: Med Phys. 2021 Jul 20;48(9):5202–18. doi: 10.1002/mp.15062 (PMC12086754; doi:10.1002/mp.15062)
Supplement: Supplementary file 1 — Supplementary Material [file MP-48-5202-s001.pdf]

Appendix A: X-ray spectra

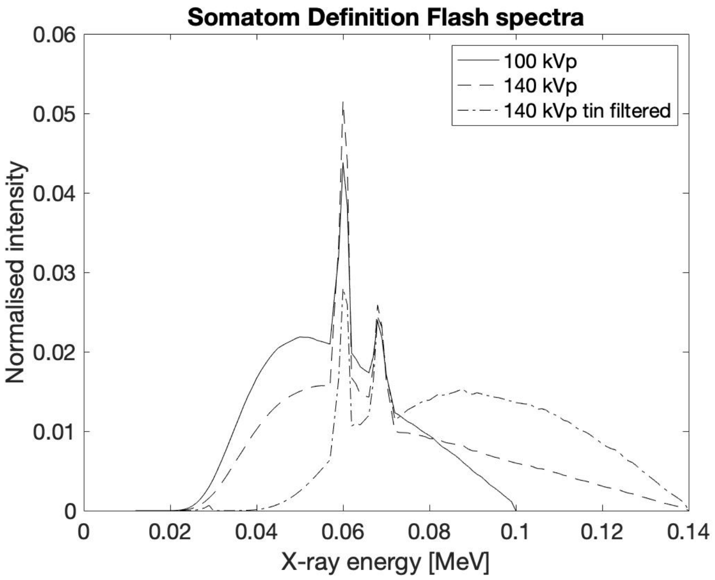

(a)

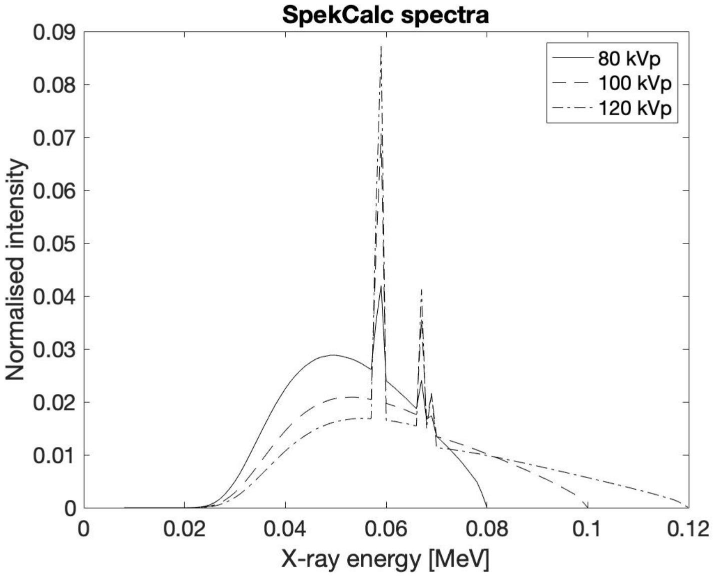

(b)

FIG. 1: Spectra used to calculate theoretical CT numbers. (a) Spectra resembling those from the Somatom Definition Flash dual-source dual-energy scanner. Those spectra were used throughout the study to calculate SECT and DECT based CT numbers. (b) Additional simulated spectra from the *SpekCalc* software, used to cover the wide range of spectra used for CT calibration in clinical proton therapy.

Appendix B: Virtual phantom

TABLE I: Structures and assigned tissues used to generate the three virtual phantoms from patient data. For the Ewing's sarcoma patient, the tissue compositions and densities are taken from the 10-year old male paediatric phantom. For the salivary sarcoma patient, the tissue compositions and densities were taken from a 15-year old male. For the Glioma patient, the tissue compositions and densities were taken from a 5-year old male. When multiple tissues are listed for the same structure, k-means clustering was used on the original CT numbers to assign the tissues.

Ewing's sarcoma patient (11.5 years)	Assigned tissues from 10-year old male
Bladder	Urinary bladder wall, urine
Body	Oral mucosa, adipose tissue, air inside the body
Bones	Mineral bone, pelvis spongiosa
Bowel space	Colon wall and rectum, adipose tissue, air inside the body
Femoral head left	Mineral bone, femora upper half spongiosa
Femoral head right	Mineral bone, femora upper half spongiosa
Femur left	Mineral bone, femora upper half spongiosa, humeri and femora upper half medullary cavity
Femur right	Mineral bone, femora upper half spongiosa, humeri femora upper half medullary cavity
Growth plate left	Cartilage
Growth plate right	Cartilage
Rectum	Colon wall and rectum
Skin	Skin
Testicle left	Testes
Testicle right	Testes
**Salivary sarcoma patient (18 years)**	**Assigned tissues from 15-year old male**
Body	Teeth, mineral bone, cranium spongiosa, oral mucosa, adipose tissue, air inside body
Brain	Brain
Brain stem	Brain
Cerebellum	Brain
Esophagus	Oesophagus wall
Hypothalamus	Brain
Lacrimal gland left	Adrenal glands
Lacrimal gland right	Adrenal glands
Larynx	Cartilage, air inside the body
Lens left	Eye lens and bulb
Lens right	Eye lens and bulb
Mandible	Mineral bone, cranium spongiosa
Masticator	Oral mucosa
Nasopharynx	Generic soft tissue
Optic chiasm	Brain
Optic nerve left	Brain
Optic nerve right	Brain
Oral cavity	Oral mucosa
Parotid left	Generic soft tissue
Pharyngeal constrictor	Oral mucosa
Pituitary gland	Generic soft tissue
Retina left	Eye lens and bulb
Retina right	Eye lens and bulb
Spinal chord	Brain
Submandibular gland left	Breast glandular tissue
Submandibular gland	Breast glandular tissue
Temporomandibular joint left	Mineral bone, cranium spongiosa, cartilage
Temporomandibular joint right	Mineral bone, cranium spongiosa, cartilage
Thyroid	Thyroid
**Glioma patient (5 years)**	**Assigned tissues from 5-year old male**
Body	Teeth, mineral bone, cranium spongiosa, oral mucosa, brain, adipose tissue, air inside body
Skin	Skin
Brain stem	Brain
Lens left	Eye lens and bulb
Lens right	Eye lens and bulb
Optic nerve left	Brain
Optic nerve right	Brain
Globe left	Eye lens and bulb
Globe right	Eye lens and bulb
Optic chiasm	Brain
Pituitary	Generic soft tissue
Spinal chord	Brain
Cochlea right	Mineral bone, cranium spongiosa, air inside body
Cochlea left	Mineral bone, cranium spongiosa, air inside body

Appendix C: SECT: List of tissues, their CT numbers, theoretical and predicted RSP.

This appendix provides lists of media investigated in this study, including their CT numbers, theoretical reference RSP, RSPs estimated with SECT, and RSP errors. For the age groups newborn, 1-year old, 5-year old and 10-year old, the tissues are identical between female and male with exceptions of tissues number 42, 46 and 48. Those tissues are denoted with F/M referring to female/male tissues. For the age groups 15-year old and adult, most tissues differ in composition and density between female and male and are hence presented in separate tables.

TABLE II: CT numbers, reference (RSP_{ref}) and predicted RSP (RSP_{SECT}) values, and RSP errors for tissue compositions in newborns.

ID	Tissue type	CT number [HU]	RSP_{ref}	RSP_{SECT}	Δ RSP_{SECT} [%]
1	Teeth	1814.44	1.41	1.57	11.96
2	Mineral bone	1382.95	1.49	1.45	-2.68
3	Humeri, upper half, spongiosa	593.18	1.24	1.22	-1.77
4	Humeri, lower half, spongiosa	277.29	1.31	1.13	-13.97
5	Ulnae and radii, spongiosa	277.29	1.31	1.13	-13.97
6	Wrists and hand, spongiosa	215.72	1.25	1.11	-11.05
7	Clavicles, spongiosa	570.38	1.18	1.21	2.31
8	Cranium, spongiosa	862.78	1.34	1.30	-2.89
9	Femora, upper half, spongiosa	593.18	1.24	1.22	-1.77
10	Femora, lower half, spongiosa	593.18	1.24	1.22	-1.77
11	Tibiae, fibulae, patellae, spongiosa	564.32	1.25	1.21	-2.87
12	Ankles and foot, spongiosa	516.39	1.18	1.20	1.31
13	Mandible, spongiosa	460.61	1.19	1.18	-1.14
14	Pelvis, spongiosa	532.23	1.19	1.20	0.65
15	Ribs, spongiosa	456.16	1.19	1.18	-1.24
16	Scapulae, spongiosa	558.82	1.19	1.21	1.70
17	Cervical spine, spongiosa	570.98	1.28	1.21	-5.58
18	Thoracic spine, spongiosa	593.73	1.29	1.22	-5.81
19	Lumbar spine, spongiosa	541.83	1.25	1.20	-3.85
20	Sacrum, spongiosa	541.83	1.25	1.20	-3.85
21	Sternum, spongiosa	517.60	1.18	1.20	1.26
22	Humeri, femora, upper half, medullary cavity	255.53	0.98	1.12	14.58
23	Humeri, femora, lower half, medullary cavity	209.35	0.99	1.11	12.00
24	Ulnae and radii, medullary cavity	6.59	1.03	1.03	-0.32
25	Tibiae, fibulae, patellae, medullary cavity	255.53	0.98	1.12	14.58
26	Cartilage	119.47	1.08	1.07	-0.61
27	Skin	93.99	1.09	1.06	-2.79
28	Blood vessels	60.70	1.05	1.05	-0.13
29	Oral mucosa	24.29	1.02	1.04	1.15
30	Liver	34.76	1.03	1.04	0.75
31	Pancreas	20.66	1.03	1.03	0.72
32	Brain	33.53	1.03	1.04	1.25
33	Heart wall	36.83	1.03	1.04	0.62
34	Eye lens and bulb	24.89	1.04	1.04	-0.22
35	Kidney tissue	30.97	1.02	1.04	1.43
36	Stomach wall	20.42	1.02	1.03	0.91
37	Small intestine wall	20.27	1.02	1.03	0.92
38	Colon wall, rectum	20.27	1.02	1.03	0.92
39	Spleen	39.31	1.03	1.04	0.98
40	Thyroid	78.94	1.04	1.06	1.36
41	Urindary bladder wall	41.61	1.03	1.04	0.78
42F	Ovaries	31.18	1.05	1.04	-0.99
42M	Testes	31.14	1.05	1.04	-0.99
43	Adrenal glands	11.27	1.03	1.03	0.28
44	Oesophagus wall	14.51	1.03	1.03	0.56
45	Generic soft tissue	10.96	1.03	1.03	0.28
46F	Uterus and cervix	10.96	1.03	1.03	0.28
46M	Prostate	11.10	1.03	1.03	0.27
47	Lymphatic nodes	11.50	1.03	1.03	0.19
48F	Breast glandular tissue	-5.12	1.03	1.02	-0.31
48M	Breast glandular tissue	-4.94	1.03	1.02	-0.28
49	Adipose tissue	-34.91	1.00	1.01	1.42
50	Lung tissue	-412.49	0.59	0.62	6.41
51	GI tract contents	20.19	1.02	1.03	1.15
52	Urine	12.46	1.00	1.03	2.73

TABLE III: CT numbers, reference (RSP_{ref}) and predicted (RSP_{SECT}) RSP values, and RSP errors for tissue compositions in 1-year old children.

ID	Tissue type	CT number [HU]	RSP_{ref}	RSP_{SECT}	Δ RSP_{SECT} [%]
1	Teeth	1814.44	1.41	1.57	11.96
2	Mineral bone	1441.68	1.49	1.47	-1.70
3	Humeri, upper half, spongiosa	743.99	1.28	1.26	-1.42
4	Humeri, lower half, spongiosa	327.28	1.37	1.14	-16.70
5	Ulnae and radii, spongiosa	375.49	1.42	1.16	-18.65
6	Wrists and hand, spongiosa	245.54	1.29	1.12	-13.16
7	Clavicles, spongiosa	450.57	1.26	1.18	-6.60
8	Cranium, spongiosa	897.42	1.33	1.31	-1.93
9	Femora, upper half, spongiosa	710.51	1.26	1.25	-0.76
10	Femora, lower half, spongiosa	690.11	1.22	1.25	2.06
11	Tibiae, fibulae, patellae, spongiosa	739.93	1.25	1.26	0.55
12	Ankles and foot, spongiosa	584.62	1.20	1.22	1.02
13	Mandible, spongiosa	849.93	1.32	1.29	-1.94
14	Pelvis, spongiosa	466.52	1.16	1.18	2.16
15	Ribs, spongiosa	483.35	1.20	1.19	-0.98
16	Scapulae, spongiosa	489.02	1.24	1.19	-4.30
17	Cervical spine, spongiosa	434.74	1.16	1.17	1.16
18	Thoracic spine, spongiosa	422.49	1.11	1.17	5.13
19	Lumbar spine, spongiosa	558.39	1.21	1.21	-0.22
20	Sacrum, spongiosa	481.22	1.23	1.19	-3.63
21	Sternum, spongiosa	504.81	1.19	1.19	0.07
22	Humeri, femora, upper half, medullary cavity	309.76	0.96	1.14	17.80
23	Humeri, femora, lower half, medullary cavity	248.63	0.98	1.12	14.32
24	Ulnae and radii, medullary cavity	-3.19	1.03	1.03	-0.45
25	Tibiae, fibulae, patellae, medullary cavity	256.92	0.97	1.12	15.16
26	Cartilage	118.18	1.08	1.07	-0.66
27	Skin	81.29	1.09	1.06	-3.14
28	Blood vessels	60.70	1.05	1.05	-0.13
29	Oral mucosa	25.32	1.02	1.04	1.31
30	Liver	43.08	1.04	1.04	-0.13
31	Pancreas	20.93	1.03	1.03	0.73
32	Brain	30.66	1.03	1.04	0.97
33	Heart wall	35.56	1.04	1.04	0.50
34	Eye lens and bulb	24.89	1.04	1.04	-0.22
35	Kidney	36.29	1.03	1.04	0.68
36	Stomach wall	21.62	1.03	1.03	0.88
37	Small intestine wall	21.62	1.03	1.03	0.88
38	Colon wall, rectum	21.62	1.03	1.03	0.88
39	Spleen	56.85	1.05	1.05	-0.42
40	Thyroid	79.98	1.04	1.06	1.29
41	Urindary bladder wall	41.70	1.03	1.04	0.78
42F	Ovaries	34.53	1.05	1.04	-0.97
42M	Testes	47.34	1.05	1.04	-0.10
43	Adrenal glands	16.10	1.03	1.03	0.31
44	Oesophagus wall	16.14	1.03	1.03	0.43
45	Generic soft tissue	15.01	1.03	1.03	0.22
46F	Uterus and cervix	7.84	1.03	1.03	-0.36
46M	Prostate	15.10	1.03	1.03	0.23
47	Lymphatic nodes	14.54	1.03	1.03	0.18
48F	Breast glandular tissue	-23.31	1.04	1.02	-2.26
48M	Breast glandular tissue	-28.38	1.04	1.02	-2.43
49	Adipose tissue	-87.84	0.98	0.99	1.08
50	Lung tissue	-607.78	0.39	0.41	4.18
51	GI tract contents	20.19	1.02	1.03	1.15
52	Urine	12.46	1.00	1.03	2.73

TABLE IV: CT numbers, reference (RSP_{ref}) and predicted (RSP_{SECT}) RSP values, and RSP errors for tissue compositions in 5-year old children.

ID	Tissue type	CT number [HU]	RSP_{ref}	RSP_{SECT}	Δ RSP_{SECT} [%]
1	Teeth	1814.44	1.41	1.57	11.96
2	Mineral bone	1595.37	1.52	1.51	-0.67
3	Humeri, upper half, spongiosa	489.60	1.26	1.19	-5.55
4	Humeri, lower half, spongiosa	241.70	1.29	1.12	-13.55
5	Ulnae and radii, spongiosa	220.79	1.27	1.11	-12.69
6	Wrists and hand, spongiosa	187.99	1.24	1.10	-11.45
7	Clavicles, spongiosa	400.21	1.20	1.16	-3.07
8	Cranium, spongiosa	775.27	1.29	1.27	-1.64
9	Femora, upper half, spongiosa	594.97	1.23	1.22	-1.06
10	Femora, lower half, spongiosa	519.14	1.21	1.20	-0.65
11	Tibiae, fibulae, patellae, spongiosa	484.84	1.16	1.19	2.36
12	Ankles and foot, spongiosa	402.59	1.21	1.16	-4.18
13	Mandible, spongiosa	695.50	1.27	1.25	-1.38
14	Pelvis, spongiosa	337.33	1.16	1.14	-1.49
15	Ribs, spongiosa	387.88	1.20	1.16	-3.81
16	Scapulae, spongiosa	493.77	1.21	1.19	-1.71
17	Cervical spine, spongiosa	469.60	1.19	1.18	-0.36
18	Thoracic spine, spongiosa	415.62	1.13	1.17	3.58
19	Lumbar spine, spongiosa	458.72	1.19	1.18	-0.62
20	Sacrum, spongiosa	335.57	1.13	1.14	1.68
21	Sternum, spongiosa	380.97	1.12	1.16	3.38
22	Humeri, femora, upper half, medullary cavity	167.78	0.99	1.09	10.34
23	Humeri, femora, lower half, medullary cavity	236.15	0.98	1.11	14.05
24	Ulnae and radii, medullary cavity	-24.69	1.02	1.02	-0.03
25	Tibiae, fibulae, patellae, medullary cavity	113.00	0.99	1.07	8.15
26	Cartilage	118.18	1.08	1.07	-0.66
27	Skin	81.24	1.09	1.06	-3.13
28	Blood vessels	60.70	1.05	1.05	-0.13
29	Oral mucosa	25.42	1.02	1.04	1.32
30	Liver	43.55	1.04	1.04	-0.09
31	Pancreas	21.20	1.03	1.03	0.75
32	Brain	40.63	1.04	1.04	0.39
33	Heart wall	35.53	1.03	1.04	0.51
34	Eye lens and bulb	24.89	1.04	1.04	-0.22
35	Kidney tissue	36.38	1.03	1.04	0.72
36	Stomach wall	21.57	1.03	1.03	0.87
37	Small intestine wall	21.57	1.03	1.03	0.87
38	Colon wall, rectum	21.57	1.03	1.03	0.87
39	Spleen	56.78	1.05	1.05	-0.41
40	Thyroid	79.98	1.04	1.06	1.29
41	UrindarBladderWall	41.70	1.03	1.04	0.78
42F	Ovaries	34.81	1.05	1.04	-0.95
42M	Testes	47.34	1.05	1.04	-0.10
43	Adrenal glands	16.23	1.03	1.03	0.44
44	Oesophagus wall	16.77	1.03	1.03	0.49
45	Generic soft tissue	15.82	1.03	1.03	0.29
46F	Uterus and cervix	8.55	1.03	1.03	-0.18
46M	Prostate	15.88	1.03	1.03	0.28
47	Lymphatic nodes	14.73	1.03	1.03	0.20
48F	Breast glandular tissue	-21.43	1.04	1.02	-1.99
48M	Breast glandular tissue	-26.34	1.04	1.02	-2.16
49	Adipose tissue	-87.67	0.98	0.99	1.09
50	Lung tissue	-614.74	0.38	0.40	4.03
51	GI tract contents	20.19	1.02	1.03	1.15
52	Urine	12.46	1.00	1.03	2.73

TABLE V: CT numbers, reference (RSP_{ref}) and predicted (RSP_{SECT}) RSP values, and RSP errors for tissue compositions in 10-year old children.

ID	Tissue type	CT number [HU]	RSP_{ref}	RSP_{SECT}	Δ RSP_{SECT} [%]
1	Teeth	2974.33	1.99	1.91	-3.70
2	Mineral bone	1736.66	1.56	1.55	-0.54
3	Humeri, upper half, spongiosa	382.44	1.20	1.16	-3.75
4	Humeri, lower half, spongiosa	154.12	1.21	1.09	-10.27
5	Ulnae and radii, spongiosa	159.84	1.23	1.09	-11.49
6	Wrists and hand, spongiosa	81.06	1.16	1.06	-8.51
7	Clavicles, spongiosa	223.07	1.12	1.11	-0.68
8	Cranium, spongiosa	600.20	1.24	1.22	-1.19
9	Femora, upper half, spongiosa	459.42	1.19	1.18	-1.15
10	Femora, lower half, spongiosa	425.83	1.17	1.17	-0.30
11	Tibiae, fibulae, patellae, spongiosa	355.90	1.11	1.15	3.27
12	Ankles and foot, spongiosa	282.54	1.14	1.13	-0.58
13	Mandible, spongiosa	520.61	1.21	1.20	-0.86
14	Pelvis, spongiosa	167.65	1.12	1.09	-2.32
15	Ribs, spongiosa	187.66	1.13	1.10	-2.38
16	Scapulae, spongiosa	312.76	1.15	1.14	-1.23
17	Cervical spine, spongiosa	265.48	1.13	1.12	-0.47
18	Thoracic spine, spongiosa	270.98	1.08	1.13	3.73
19	Lumbar spine, spongiosa	284.14	1.14	1.13	-0.88
20	Sacrum, spongiosa	173.96	1.06	1.09	3.45
21	Sternum, spongiosa	220.90	1.06	1.11	4.83
22	Humeri, femora, upper half, medullary cavity	130.31	0.99	1.08	9.24
23	Humeri, femora, lower half, medullary cavity	151.71	0.98	1.09	10.40
24	Ulnae and radii, medullary cavity	-52.94	1.01	1.01	-0.16
25	Tibiae, fibulae, patellae, medullary cavity	89.63	0.98	1.06	8.45
26	Cartilage	118.53	1.08	1.07	-0.66
27	Skin	81.14	1.09	1.06	-3.13
28	Blood vessels	60.70	1.05	1.05	-0.13
29	Oral mucosa	25.42	1.02	1.04	1.32
30	Liver	43.55	1.04	1.04	-0.09
31	Pancreas	21.20	1.03	1.03	0.75
32	Brain	40.63	1.04	1.04	0.39
33	Heart wall	35.53	1.03	1.04	0.51
34	Eye lens and bulb	24.89	1.04	1.04	-0.22
35	Kidney tissue	36.38	1.03	1.04	0.72
36	Stomach wall	21.57	1.03	1.03	0.87
37	Small intestine wall	21.57	1.03	1.03	0.87
38	Colon wall, rectum	21.57	1.03	1.03	0.87
39	Spleen	56.78	1.05	1.05	-0.41
40	Thyroid	79.98	1.04	1.06	1.29
41	Urindary bladder wall	41.70	1.03	1.04	0.78
42F	Ovaries	34.81	1.05	1.04	-0.95
42M	Testes	47.34	1.05	1.04	-0.10
43	Adrenal glands	16.23	1.03	1.03	0.44
44	Oesophagus wall	16.77	1.03	1.03	0.49
45	Generic soft tissue	16.10	1.03	1.03	0.31
46F	Uterus and cervix	8.92	1.03	1.03	-0.16
46M	Prostate	16.25	1.03	1.03	0.31
47	Lymphatic nodes	14.73	1.03	1.03	0.20
48F	Breast glandular tissue	-20.79	1.04	1.02	-1.94
48M	Breast glandular tissue	-25.14	1.04	1.02	-2.07
49	Adipose tissue	-87.67	0.98	0.99	1.09
50	Lung tissue	-565.58	0.43	0.45	5.38
51	GI tract contents	20.19	1.02	1.03	1.15
52	Urine	12.46	1.00	1.03	2.73

TABLE VI: CT numbers, reference (RSP_{ref}) and predicted (RSP_{SECT}) RSP values, and RSP errors for tissue compositions in a 15-year old female.

ID	Tissue type	CT number [HU]	RSP_{ref}	RSP_{SECT}	Δ RSP_{SECT} [%]
1	Teeth	4117.17	2.56	1.95	-23.77
2	Mineral bone	1843.76	1.60	1.58	-1.01
3	Humeri, upper half, spongiosa	234.40	1.12	1.11	-0.31
4	Humeri, lower half, spongiosa	99.67	1.17	1.07	-8.94
5	Ulnae and radii, spongiosa	103.81	1.18	1.07	-9.77
6	Wrists and hand, spongiosa	20.56	1.10	1.03	-6.15
7	Clavicles, spongiosa	228.32	1.13	1.11	-1.26
8	Cranium, spongiosa	501.09	1.21	1.19	-1.28
9	Femora, upper half, spongiosa	272.57	1.15	1.13	-2.04
10	Femora, lower half, spongiosa	358.16	1.18	1.15	-2.64
11	Tibiae, fibulae, patellae, spongiosa	222.57	1.07	1.11	4.24
12	Ankles and foot, spongiosa	207.02	1.06	1.11	4.30
13	Mandible, spongiosa	441.41	1.19	1.17	-0.91
14	Pelvis, spongiosa	110.77	1.09	1.07	-2.03
15	Ribs, spongiosa	165.14	1.10	1.09	-0.85
16	Scapulae, spongiosa	367.56	1.22	1.15	-5.47
17	Cervical spine, spongiosa	217.65	1.12	1.11	-1.33
18	Thoracic spine, spongiosa	325.37	1.08	1.14	6.10
19	Lumbar spine, spongiosa	214.66	1.08	1.11	2.79
20	Sacrum, spongiosa	116.92	1.05	1.07	2.55
21	Sternum, spongiosa	153.66	1.08	1.09	0.85
22	Humeri, femora, upper half, medullary cavity	171.92	0.97	1.09	12.65
23	Humeri, femora, lower half, medullary cavity	57.46	0.99	1.05	6.19
24	Ulnae and radii, medullary cavity	-69.17	1.00	1.00	-0.14
25	Tibiae, fibulae, patellae, medullary cavity	104.96	0.96	1.07	10.64
26	Cartilage	119.47	1.08	1.07	-0.61
27	Skin	81.29	1.09	1.06	-3.14
28	Blood vessels	60.70	1.05	1.05	-0.13
29	Oral mucosa	15.46	1.01	1.03	1.93
30	Liver	43.53	1.04	1.04	-0.11
31	Pancreas	11.04	1.02	1.03	1.35
32	Brain	40.66	1.04	1.04	0.37
33	Heart wall	35.37	1.03	1.04	0.52
34	Eye lens and bulb	24.89	1.04	1.04	-0.22
35	Kidney tissue	36.29	1.03	1.04	0.71
36	Stomach wall	21.62	1.03	1.03	0.88
37	Small intestine wall	21.51	1.03	1.03	0.88
38	Colon wall, rectum	21.51	1.03	1.03	0.88
39	Spleen	56.78	1.05	1.05	-0.41
40	Thyroid	79.98	1.04	1.06	1.29
41	Urindary bladder wall	41.70	1.03	1.04	0.78
42	Ovaries	34.72	1.05	1.04	-0.96
43	Adrenal glands	6.00	1.02	1.03	1.02
44	Oesophagus wall	16.42	1.03	1.03	0.45
45	Generic soft tissue	5.25	1.02	1.03	0.83
46	Uterus and cervix	8.02	1.03	1.03	-0.35
47	Lymphatic nodes	4.79	1.02	1.03	0.80
48	Breast glandular tissue	-23.02	1.04	1.02	-2.22
49	Adipose tissue	-106.78	0.96	0.97	1.39
50	Lung tissue	-694.71	0.30	0.31	2.19
51	GI tract contents	10.29	1.01	1.03	1.76
52	Urine	12.46	1.00	1.03	2.73

TABLE VII: CT numbers, reference (RSP_{ref}) and predicted (RSP_{SECT}) RSP values, and RSP errors for tissue compositions in a 15-year old male.

ID	Tissue type	CT number [HU]	RSP_{ref}	RSP_{SECT}	Δ RSP_{SECT} [%]
1	Teeth	4117.17	2.56	1.95	-23.77
2	Mineral bone	1843.76	1.60	1.58	-1.01
3	Humeri, upper half, spongiosa	234.00	1.12	1.11	-0.34
4	Humeri, lower half, spongiosa	100.37	1.17	1.07	-9.12
5	Ulnae and radii, spongiosa	104.13	1.18	1.07	-9.86
6	Wrists and hand, spongiosa	21.21	1.10	1.03	-6.22
7	Clavicles, spongiosa	229.81	1.13	1.11	-1.25
8	Cranium, spongiosa	502.89	1.20	1.19	-0.89
9	Femora, upper half, spongiosa	275.37	1.15	1.13	-1.97
10	Femora, lower half, spongiosa	358.02	1.18	1.15	-2.67
11	Tibiae, fibulae, patellae, spongiosa	224.92	1.07	1.11	4.32
12	Ankles and foot, spongiosa	207.96	1.06	1.11	4.20
13	Mandible, spongiosa	446.39	1.19	1.18	-0.78
14	Pelvis, spongiosa	113.79	1.09	1.07	-2.03
15	Ribs, spongiosa	164.74	1.10	1.09	-1.00
16	Scapulae, spongiosa	369.71	1.22	1.15	-5.54
17	Cervical spine, spongiosa	219.14	1.12	1.11	-1.32
18	Thoracic spine, spongiosa	330.91	1.08	1.14	6.24
19	Lumbar spine, spongiosa	216.87	1.08	1.11	2.83
20	Sacrum, spongiosa	118.69	1.05	1.07	2.46
21	Sternum, spongiosa	155.45	1.08	1.09	0.76
22	Humeri, femora, upper half, medullary cavity	173.58	0.97	1.09	12.84
23	Humeri, femora, lower half, medullary cavity	57.11	0.99	1.05	6.17
24	Ulnae and radii, medullary cavity	-69.45	1.00	1.00	-0.17
25	Tibiae, fibulae, patellae, medullary cavity	107.12	0.96	1.07	10.84
26	Cartilage	118.53	1.08	1.07	-0.66
27	Skin	81.24	1.09	1.06	-3.13
28	Blood vessels	60.70	1.05	1.05	-0.13
29	Oral mucosa	25.42	1.02	1.04	1.32
30	Liver	43.78	1.04	1.04	-0.08
31	Pancreas	21.27	1.03	1.03	0.77
32	Brain	40.63	1.04	1.04	0.39
33	Heart wall	35.47	1.03	1.04	0.52
34	Eye lens and bulb	24.89	1.04	1.04	-0.22
35	Kidney tissue	36.47	1.03	1.04	0.72
36	Stomach wall	21.73	1.03	1.03	0.87
37	Small intestine wall	21.61	1.03	1.03	0.87
38	Colon wall, rectum	21.61	1.03	1.03	0.87
39	Spleen	61.61	1.05	1.05	-0.09
40	Thyroid	80.02	1.04	1.06	1.29
41	Urindary bladder wall	41.70	1.03	1.04	0.78
42	Testes	47.34	1.05	1.04	-0.10
43	Adrenal glands	16.79	1.03	1.03	0.48
44	Oesophagus wall	17.36	1.03	1.03	0.55
45	Generic soft tissue	15.99	1.03	1.03	0.43
46	Prostate	15.99	1.03	1.03	0.43
47	Lymphatic nodes	14.82	1.03	1.03	0.21
48	Breast glandular tissue	-23.82	1.04	1.02	-1.98
49	Adipose tissue	-106.66	0.96	0.97	1.40
50	Lung tissue	-640.65	0.36	0.37	3.80
51	GI tract contents	20.19	1.02	1.03	1.15
52	Urine	12.46	1.00	1.03	2.73

TABLE VIII: CT numbers, reference (RSP_{ref}) and predicted (RSP_{SECT}) RSP values, and RSP errors for tissue compositions in an adult female.

ID	Tissue type	CT number [HU]	RSP_{ref}	RSP_{SECT}	Δ RSP_{SECT} [%]
1	Teeth	4102.06	2.34	1.95	-16.82
2	Mineral bone	2095.04	1.70	1.66	-2.35
3	Humeri, upper half, spongiosa	393.25	1.17	1.16	-1.02
4	Humeri, lower half, spongiosa	216.00	1.10	1.11	1.11
5	Ulnae and radii, spongiosa	216.00	1.10	1.11	1.11
6	Wrists and hand, spongiosa	216.00	1.10	1.11	1.11
7	Clavicles, spongiosa	338.70	1.12	1.14	2.26
8	Cranium, spongiosa	396.56	1.11	1.16	4.36
9	Femora, upper half, spongiosa	142.34	1.13	1.08	-4.17
10	Femora, lower half, spongiosa	216.00	1.10	1.11	1.11
11	Tibiae, fibulae, patellae, spongiosa	216.00	1.10	1.11	1.11
12	Ankles and foot, spongiosa	216.00	1.10	1.11	1.11
13	Mandible, spongiosa	424.13	1.19	1.17	-2.08
14	Pelvis, spongiosa	212.24	1.11	1.11	-0.26
15	Ribs, spongiosa	233.10	1.15	1.11	-3.49
16	Scapulae, spongiosa	302.68	1.17	1.13	-2.72
17	Cervical spine, spongiosa	154.67	1.03	1.09	5.42
18	Thoracic spine, spongiosa	130.08	1.07	1.08	1.05
19	Lumbar spine, spongiosa	261.10	1.08	1.12	3.55
20	Sacrum, spongiosa	45.60	1.03	1.04	1.29
21	Sternum, spongiosa	85.93	1.04	1.06	2.40
22	Humeri, femora, upper half, medullary cavity	-71.79	1.00	1.00	-0.61
23	Humeri, femora, lower half, medullary cavity	-71.79	1.00	1.00	-0.61
24	Ulnae and radii, medullary cavity	-71.79	1.00	1.00	-0.61
25	Tibiae, fibulae, patellae, medullary cavity	-71.79	1.00	1.00	-0.61
26	Cartilage	119.47	1.08	1.07	-0.61
27	Skin	71.56	1.08	1.05	-2.60
28	Blood vessels	60.70	1.05	1.05	-0.13
29	Oral mucosa	45.33	1.04	1.04	0.13
30	Liver	45.98	1.04	1.04	0.21
31	Pancreas	40.88	1.05	1.04	-0.43
32	Brain	50.67	1.05	1.05	-0.21
33	Heart wall	45.17	1.04	1.04	-0.09
34	Eye lens and bulb	26.84	1.04	1.04	-0.21
35	Kidney tissue	46.41	1.04	1.04	0.12
36	Stomach wall	31.42	1.04	1.04	0.28
37	Small intestine wall	31.42	1.04	1.04	0.28
38	Colon wall, rectum	31.42	1.04	1.04	0.28
39	Spleen	36.84	1.03	1.04	0.75
40	Thyroid	69.70	1.03	1.05	1.88
41	Urindary bladder wall	41.70	1.03	1.04	0.78
42	Ovaries	38.12	1.03	1.04	0.64
43	Adrenal glands	15.99	1.03	1.03	0.43
44	Oesophagus wall	16.55	1.03	1.03	0.48
45	Generic soft tissue	15.97	1.03	1.03	0.29
46	Uterus and cervix	8.59	1.03	1.03	-0.17
47	Lymphatic nodes	31.07	1.03	1.04	1.19
48	Breast glandular tissue	-21.28	1.04	1.02	-1.96
49	Adipose tissue	-96.69	0.97	0.99	1.66
50	Lung tissue	-618.71	0.38	0.39	3.93
51	GI tract contents	30.10	1.03	1.04	0.55
52	Urine	42.54	1.03	1.04	0.90

TABLE IX: CT numbers, reference (RSP_{ref}) and predicted (RSP_{SECT}) RSP values, and RSP errors for tissue compositions in an adult male.

ID	Tissue type	CT number [HU]	RSP_{ref}	RSP_{SECT}	Δ RSP_{SECT} [%]
1	Teeth	4102.06	2.34	1.95	-16.82
2	Mineral bone	2095.04	1.70	1.66	-2.35
3	Humeri, upper half, spongiosa	400.89	1.17	1.16	-0.29
4	Humeri, lower half, spongiosa	199.25	1.10	1.10	0.58
5	Ulnae and radii, spongiosa	199.25	1.10	1.10	0.58
6	Wrists and hand, spongiosa	199.25	1.10	1.10	0.58
7	Clavicles, spongiosa	284.24	1.13	1.13	0.15
8	Cranium, spongiosa	298.94	1.13	1.13	0.21
9	Femora, upper half, spongiosa	226.85	1.11	1.11	0.44
10	Femora, lower half, spongiosa	199.25	1.10	1.10	0.58
11	Tibiae, fibulae, patellae, spongiosa	199.25	1.10	1.10	0.58
12	Ankles and foot, spongiosa	199.25	1.10	1.10	0.58
13	Mandible, spongiosa	452.34	1.18	1.18	-0.48
14	Pelvis, spongiosa	221.99	1.11	1.11	0.45
15	Ribs, spongiosa	309.76	1.14	1.14	0.05
16	Scapulae, spongiosa	355.05	1.15	1.15	-0.01
17	Cervical spine, spongiosa	60.73	1.05	1.05	-0.09
18	Thoracic spine, spongiosa	113.16	1.07	1.07	0.27
19	Lumbar spine, spongiosa	190.93	1.10	1.10	0.37
20	Sacrum, spongiosa	21.24	1.04	1.03	-0.14
21	Sternum, spongiosa	41.93	1.04	1.04	-0.12
22	Humeri, femora, upper half, medullary cavity	-71.70	1.00	1.00	-0.60
23	Humeri, femora, lower half, medullary cavity	-71.70	1.00	1.00	-0.60
24	Ulnae and radii, medullary cavity	-71.70	1.00	1.00	-0.60
25	Tibiae, fibulae, patellae, medullary cavity	-71.70	1.00	1.00	-0.60
26	Cartilage	119.47	1.08	1.07	-0.61
27	Skin	71.56	1.08	1.05	-2.60
28	Blood	60.70	1.05	1.05	-0.13
29	MuscleTissue	45.33	1.04	1.04	0.13
30	Liver	46.08	1.04	1.04	0.22
31	Pancreas	41.01	1.05	1.04	-0.42
32	Brain	50.71	1.05	1.05	-0.21
33	Heart	55.12	1.05	1.05	-0.67
34	Eyes	27.08	1.04	1.04	-0.20
35	Kidneys	46.51	1.04	1.04	0.13
36	Stomach	31.42	1.04	1.04	0.28
37	SmallIntestine	31.46	1.04	1.04	0.29
38	LargeIntestine	31.46	1.04	1.04	0.29
39	Spleen	41.58	1.03	1.04	1.07
40	Thyroid	69.73	1.03	1.05	1.88
41	UrinaryBladder	41.70	1.03	1.04	0.78
42	Testes	37.44	1.04	1.04	0.48
43	Adrenals	16.64	1.03	1.03	0.48
44	Oesophagus	17.33	1.03	1.03	0.53
45	Generic soft tissue	15.71	1.03	1.03	0.41
46	Prostate	15.71	1.03	1.03	0.41
47	Lymph	31.07	1.03	1.04	1.19
48	Breast	-24.89	1.04	1.02	-2.04
49	Adipose tissue	-96.65	0.97	0.99	1.66
50	LungTissueCompressed	-618.71	0.38	0.39	3.93
51	GI tract contents	30.10	1.03	1.04	0.55
52	Urine	42.54	1.03	1.04	0.90

Appendix D: DECT: List of tissues, their CT numbers, theoretical and predicted RSP.

This appendix provides lists of media investigated in this study, including their CT numbers (per CT energy spectrum), theoretical reference RSP, RSPs estimated with DECT, and RSP errors. For the age groups newborn, 1-year old, 5-year old and 10-year old, the tissues are identical between female and male with exceptions of tissues number 42, 46 and 48. Those tissues are denoted with F/M referring to female/male tissues. For the age groups 15-year old and adult, most tissues differ in composition and density between female and male and are hence presented in separate tables.

TABLE X: DECT: CT numbers, reference (RSP_{ref}) and predicted RSP (RSP_{DECT}) values, and RSP errors for tissue compositions in newborns.

ID	Tissue type	CT number [HU] 80 kVp	CT number [HU] 140 kVp (Sn)	RSP_{ref}	RSP_{DECT}	Δ RSP_{DECT} [%]
1	Teeth	2570.65	1076.06	1.41	1.38	-2.68
2	Mineral Bone	1862.41	914.29	1.49	1.50	0.61
3	Humeri, upper half, spongiosa	784.71	405.92	1.24	1.25	0.34
4	Humeri, lower half, spongiosa	266.14	288.04	1.31	1.31	-0.23
5	Ulnae and radii, spongiosa	266.14	288.04	1.31	1.31	-0.23
6	Wrists and hand, spongiosa	205.11	225.95	1.25	1.24	-0.23
7	Clavicles, spongiosa	779.17	366.25	1.19	1.19	0.38
8	Cranium, spongiosa	1146.83	585.10	1.34	1.34	0.35
9	Femora, upper half, spongiosa	784.71	405.92	1.24	1.25	0.34
10	Femora, lower half, spongiosa	784.71	405.92	1.24	1.25	0.34
11	Tibiae, fibulae, patellae, spongiosa	737.84	394.66	1.25	1.25	0.29
12	Ankles and foot, spongiosa	698.68	338.15	1.18	1.19	0.34
13	Mandible, spongiosa	606.91	317.51	1.19	1.20	0.19
14	Pelvis, spongiosa	716.43	352.13	1.19	1.20	0.34
15	Ribs, spongiosa	599.96	315.54	1.19	1.20	0.20
16	Scapulae, spongiosa	759.67	362.46	1.19	1.19	0.35
17	Cervical spine, spongiosa	728.33	417.07	1.29	1.29	0.19
18	Thoracic spine, spongiosa	757.63	433.46	1.30	1.30	0.23
19	Lumbar spine, spongiosa	700.39	386.78	1.25	1.26	0.23
20	Sacrum, spongiosa	700.39	386.78	1.25	1.26	0.23
21	Sternum, spongiosa	700.05	339.22	1.18	1.19	0.34
22	Humeri, femora, upper half, medullary cavity	406.47	107.95	0.98	0.98	0.34
23	Humeri, femora, lower half, medullary cavity	330.48	90.87	0.99	0.99	0.19
24	Ulnae and radii, medullary cavity	-2.20	15.06	1.03	1.03	-0.23
25	Tibiae, fibulae, patellae, medullary cavity	406.47	107.95	0.98	0.98	0.34
26	Cartilage	140.33	99.13	1.08	1.08	0.18
27	Skin	95.31	92.68	1.09	1.10	0.16
28	Blood vessels	66.69	54.81	1.05	1.05	0.07
29	Oral mucosa	25.32	23.26	1.02	1.03	0.18
30	Liver	37.28	32.26	1.03	1.03	0.11
31	Pancreas	19.27	21.98	1.03	1.03	0.05
32	Brain	37.91	29.24	1.03	1.03	0.16
33	Heart wall	38.90	34.80	1.03	1.04	0.19
34	Eye lens and bulb	19.22	30.37	1.04	1.04	0.25
35	Kidney tissue	35.49	26.53	1.02	1.03	0.15
36	Stomach wall	19.19	21.60	1.02	1.03	0.19
37	Small intestine wall	19.00	21.48	1.02	1.03	0.19
38	Colon wall, rectum	19.00	21.48	1.02	1.03	0.19
39	Spleen	44.36	34.33	1.03	1.03	0.10
40	Thyroid	94.42	60.57	1.04	1.05	0.36
41	Urinary bladder wall	46.51	36.80	1.03	1.04	0.07
42F	Ovaries	23.71	38.43	1.05	1.05	0.23
42M	Testes	23.65	38.41	1.05	1.05	0.23
43	Adrenal glands	4.20	18.14	1.03	1.03	0.25
44	Oesophagus wall	9.91	18.96	1.03	1.03	0.19
45	Generic soft tissue	3.76	17.94	1.03	1.03	0.25
46F	Uterus and cervix	3.76	17.94	1.03	1.03	0.25
46M	Prostate	3.93	18.06	1.03	1.03	0.25
47	Lymphatic nodes	4.15	18.63	1.03	1.03	0.23
48F	Breast glandular tissue	-18.19	7.58	1.03	1.03	0.04
48M	Breast glandular tissue	-17.86	7.61	1.03	1.03	0.04
49	Adipose tissue	-48.28	-21.92	1.00	1.00	0.02
50	Lung tissue	-411.98	-413.00	0.59	0.59	0.22
51	GI tract contents	21.19	19.16	1.02	1.02	-0.05
52	Urine	16.51	8.51	1.00	1.01	0.38

TABLE XI: DECT: CT numbers, reference (RSP_{ref}) and predicted RSP (RSP_{DECT}) values, and RSP errors for tissue compositions in 1-year old children.

ID	Tissue type	CT number [HU] 80 kVp	CT number [HU] 140 kVp (Sn)	RSP_{ref}	RSP_{DECT}	Δ RSP_{DECT} [%]
1	Teeth	2570.65	1076.06	1.41	1.38	-2.68
2	Mineral Bone	1951.44	943.43	1.50	1.51	0.71
3	Humeri, upper half, spongiosa	994.91	498.68	1.28	1.29	0.26
4	Humeri, lower half, spongiosa	311.64	342.40	1.37	1.37	-0.22
5	Ulnae and radii, spongiosa	359.11	391.33	1.42	1.42	-0.22
6	Wrists and hand, spongiosa	230.37	260.20	1.29	1.28	-0.22
7	Clavicles, spongiosa	557.30	346.12	1.26	1.26	-0.25
8	Cranium, spongiosa	1201.84	599.83	1.34	1.34	0.27
9	Femora, upper half, spongiosa	953.13	473.31	1.26	1.27	0.29
10	Femora, lower half, spongiosa	943.43	442.46	1.22	1.23	0.26
11	Tibiae, fibulae, patellae, spongiosa	1002.56	483.18	1.26	1.26	0.23
12	Ankles and foot, spongiosa	791.50	382.36	1.21	1.21	0.27
13	Mandible, spongiosa	1136.55	569.74	1.32	1.33	0.27
14	Pelvis, spongiosa	635.54	301.26	1.16	1.16	0.25
15	Ribs, spongiosa	639.56	330.60	1.20	1.20	0.18
16	Scapulae, spongiosa	625.71	355.31	1.24	1.24	0.01
17	Cervical spine, spongiosa	585.83	286.99	1.16	1.16	0.18
18	Thoracic spine, spongiosa	591.78	256.96	1.11	1.12	0.26
19	Lumbar spine, spongiosa	747.63	373.36	1.21	1.22	0.25
20	Sacrum, spongiosa	619.58	345.87	1.23	1.23	0.02
21	Sternum, spongiosa	675.73	337.68	1.19	1.20	0.22
22	Humeri, femora, upper half, medullary cavity	496.80	126.91	0.97	0.97	0.27
23	Humeri, femora, lower half, medullary cavity	396.82	103.73	0.98	0.98	0.13
24	Ulnae and radii, medullary cavity	-15.22	8.44	1.03	1.03	-0.22
25	Tibiae, fibulae, patellae, medullary cavity	411.47	105.82	0.97	0.98	0.26
26	Cartilage	138.34	98.53	1.08	1.08	0.18
27	Skin	77.61	84.84	1.09	1.09	0.09
28	Blood vessels	66.69	54.81	1.05	1.05	0.07
29	Oral mucosa	28.14	22.54	1.02	1.02	0.04
30	Liver	44.31	41.85	1.04	1.04	0.00
31	Pancreas	19.63	22.15	1.03	1.03	0.05
32	Brain	34.05	27.32	1.03	1.03	-0.08
33	Heart wall	37.46	33.68	1.03	1.04	0.04
34	Eye lens and bulb	19.22	30.37	1.04	1.04	0.25
35	Kidney tissue	39.12	33.49	1.03	1.03	0.07
36	Stomach wall	20.61	22.59	1.03	1.03	0.18
37	Small intestine wall	20.61	22.59	1.03	1.03	0.18
38	Colon wall, rectum	20.61	22.59	1.03	1.03	0.18
39	Spleen	60.08	53.68	1.05	1.05	0.10
40	Thyroid	95.52	61.55	1.04	1.05	0.35
41	Urinary bladder wall	46.66	36.85	1.03	1.04	0.07
42F	Ovaries	29.80	39.08	1.05	1.05	-0.12
42M	Testes	49.30	45.41	1.05	1.05	0.13
43	Adrenal glands	12.24	19.82	1.03	1.03	-0.10
44	Oesophagus wall	12.70	19.45	1.03	1.03	-0.07
45	Generic soft tissue	10.48	19.37	1.03	1.03	-0.11
46F	Uterus and cervix	-1.28	16.67	1.03	1.03	-0.13
46M	Prostate	10.62	19.42	1.03	1.03	-0.11
47	Lymphatic nodes	9.76	19.16	1.03	1.03	-0.12
48F	Breast glandular tissue	-49.15	1.81	1.04	1.04	0.08
48M	Breast glandular tissue	-55.91	-1.63	1.04	1.04	0.06
49	Adipose tissue	-115.57	-60.89	0.98	0.98	0.12
50	Lung tissue	-606.29	-609.24	0.39	0.39	0.11
51	GI tract contents	21.19	19.16	1.02	1.02	-0.05
52	Urine	16.51	8.51	1.00	1.01	0.38

TABLE XII: DECT: CT numbers, reference (RSP_{ref}) and predicted RSP (RSP_{DECT}) values, and RSP errors for tissue compositions in 5-year old children.

ID	Tissue type	CT number [HU] 80 kVp	CT number [HU] 140 kVp (Sn)	RSP_{ref}	RSP_{DECT}	Δ RSP_{DECT} [%]
1	Teeth	2570.65	1076.06	1.41	1.38	-2.68
2	Mineral Bone	2172.97	1030.79	1.53	1.54	0.76
3	Humeri, upper half, spongiosa	619.58	362.43	1.26	1.26	-0.29
4	Humeri, lower half, spongiosa	223.06	259.74	1.29	1.29	-0.23
5	Ulnae and radii, spongiosa	201.59	239.38	1.27	1.27	-0.23
6	Wrists and hand, spongiosa	167.66	207.68	1.24	1.24	-0.25
7	Clavicles, spongiosa	512.95	289.89	1.20	1.20	-0.25
8	Cranium, spongiosa	1037.42	518.97	1.30	1.30	0.11
9	Femora, upper half, spongiosa	793.42	400.92	1.23	1.24	0.13
10	Femora, lower half, spongiosa	692.55	349.55	1.21	1.21	-0.02
11	Tibiae, fibulae, patellae, spongiosa	663.76	309.88	1.16	1.16	0.00
12	Ankles and foot, spongiosa	509.92	297.58	1.21	1.21	-0.45
13	Mandible, spongiosa	929.58	466.64	1.27	1.27	0.12
14	Pelvis, spongiosa	436.88	239.91	1.16	1.16	-0.38
15	Ribs, spongiosa	491.20	286.78	1.21	1.20	-0.33
16	Scapulae, spongiosa	650.19	340.78	1.21	1.21	0.02
17	Cervical spine, spongiosa	625.58	317.03	1.19	1.19	0.02
18	Thoracic spine, spongiosa	574.65	260.11	1.13	1.13	0.06
19	Lumbar spine, spongiosa	608.92	311.83	1.19	1.19	0.01
20	Sacrum, spongiosa	453.27	220.42	1.13	1.12	-0.17
21	Sternum, spongiosa	525.57	239.53	1.12	1.12	0.02
22	Humeri, femora, upper half, medullary cavity	268.18	69.57	0.99	0.99	-0.29
23	Humeri, femora, lower half, medullary cavity	379.70	95.75	0.98	0.98	-0.16
24	Ulnae and radii, medullary cavity	-40.39	-9.48	1.02	1.01	-0.23
25	Tibiae, fibulae, patellae, medullary cavity	184.32	43.19	0.99	0.98	-0.61
26	Cartilage	138.34	98.53	1.08	1.08	0.18
27	Skin	77.57	84.76	1.09	1.09	0.09
28	Blood vessels	66.69	54.81	1.05	1.05	0.07
29	Oral mucosa	28.28	22.58	1.02	1.02	0.05
30	Liver	45.05	42.06	1.04	1.04	0.01
31	Pancreas	20.06	22.28	1.03	1.03	0.06
32	Brain	44.09	37.23	1.04	1.04	-0.08
33	Heart wall	37.47	33.61	1.03	1.04	0.05
34	Eye lens and bulb	19.22	30.37	1.04	1.04	0.25
35	Kidney tissue	39.37	33.43	1.03	1.03	0.08
36	Stomach wall	20.52	22.57	1.03	1.03	0.17
37	Small intestine wall	20.52	22.57	1.03	1.03	0.17
38	Colon wall, rectum	20.52	22.57	1.03	1.03	0.17
39	Spleen	60.02	53.59	1.05	1.05	0.11
40	Thyroid	95.52	61.55	1.04	1.05	0.35
41	Urinary bladder wall	46.66	36.85	1.03	1.04	0.07
42F	Ovaries	30.25	39.21	1.05	1.05	-0.11
42M	Testes	49.30	45.41	1.05	1.05	0.13
43	Adrenal glands	12.84	19.49	1.03	1.03	-0.07
44	Oesophagus wall	13.73	19.69	1.03	1.03	-0.06
45	Generic soft tissue	11.80	19.70	1.03	1.03	-0.10
46F	Uterus and cervix	0.28	16.56	1.03	1.03	-0.10
46M	Prostate	11.84	19.77	1.03	1.03	-0.10
47	Lymphatic nodes	10.04	19.25	1.03	1.03	-0.12
48F	Breast glandular tissue	-45.73	2.18	1.04	1.04	0.07
48M	Breast glandular tissue	-52.28	-1.13	1.04	1.04	0.04
49	Adipose tissue	-115.30	-60.81	0.98	0.98	0.12
50	Lung tissue	-613.28	-616.17	0.38	0.38	0.11
51	GI tract contents	21.19	19.16	1.02	1.02	-0.05
52	Urine	16.51	8.51	1.00	1.01	0.38

TABLE XIII: DECT: CT numbers, reference (RSP_{ref}) and predicted RSP (RSP_{DECT}) values, and RSP errors for tissue compositions in 10-year old children.

ID	Tissue type	CT number [HU] 80 kVp	CT number [HU] 140 kVp (Sn)	RSP_{ref}	RSP_{DECT}	Δ RSP_{DECT} [%]
1	Teeth	4042.20	1931.66	2.00	1.94	-2.68
2	Mineral Bone	2369.72	1117.89	1.57	1.58	0.73
3	Humeri, upper half, spongiosa	486.56	280.55	1.20	1.19	-0.68
4	Humeri, lower half, spongiosa	132.70	174.86	1.21	1.21	-0.26
5	Ulnae and radii, spongiosa	131.87	187.02	1.23	1.23	-0.01
6	Wrists and hand, spongiosa	50.86	110.42	1.15	1.16	0.08
7	Clavicles, spongiosa	284.75	162.66	1.12	1.11	-0.73
8	Cranium, spongiosa	800.53	404.31	1.24	1.24	0.02
9	Femora, upper half, spongiosa	607.14	314.95	1.19	1.19	-0.10
10	Femora, lower half, spongiosa	567.71	287.04	1.17	1.17	-0.33
11	Tibiae, fibulae, patellae, spongiosa	492.32	222.46	1.11	1.11	-0.34
12	Ankles and foot, spongiosa	369.16	197.75	1.13	1.13	-0.81
13	Mandible, spongiosa	693.25	351.78	1.21	1.21	-0.02
14	Pelvis, spongiosa	200.90	135.01	1.12	1.11	-0.97
15	Ribs, spongiosa	226.67	149.39	1.13	1.12	-0.90
16	Scapulae, spongiosa	404.85	222.63	1.15	1.15	-0.46
17	Cervical spine, spongiosa	344.39	188.22	1.13	1.12	-0.58
18	Thoracic spine, spongiosa	376.21	168.04	1.08	1.08	-0.33
19	Lumbar spine, spongiosa	368.19	201.85	1.14	1.13	-0.62
20	Sacrum, spongiosa	241.90	107.43	1.06	1.05	-0.68
21	Sternum, spongiosa	312.54	131.21	1.06	1.06	-0.39
22	Humeri, femora, upper half, medullary cavity	213.98	48.43	0.99	0.98	-0.69
23	Humeri, femora, lower half, medullary cavity	248.83	56.67	0.98	0.98	-0.68
24	Ulnae and radii, medullary cavity	-77.18	-29.39	1.01	1.01	0.06
25	Tibiae, fibulae, patellae, medullary cavity	155.85	24.80	0.98	0.97	-0.92
26	Cartilage	138.78	98.79	1.08	1.08	0.18
27	Skin	77.42	84.72	1.09	1.09	0.09
28	Blood vessels	66.69	54.81	1.05	1.05	0.07
29	Oral mucosa	28.28	22.58	1.02	1.02	0.05
30	Liver	45.05	42.06	1.04	1.04	0.01
31	Pancreas	20.06	22.28	1.03	1.03	0.06
32	Brain	44.09	37.23	1.04	1.04	-0.08
33	Heart wall	37.47	33.61	1.03	1.04	0.05
34	Eye lens and bulb	19.22	30.37	1.04	1.04	0.25
35	Kidney tissue	39.37	33.43	1.03	1.03	0.08
36	Stomach wall	20.52	22.57	1.03	1.03	0.17
37	Small intestine wall	20.52	22.57	1.03	1.03	0.17
38	Colon wall, rectum	20.52	22.57	1.03	1.03	0.17
39	Spleen	60.02	53.59	1.05	1.05	0.11
40	Thyroid	95.52	61.55	1.04	1.05	0.35
41	Urinary bladder wall	46.66	36.85	1.03	1.04	0.07
42F	Ovaries	30.25	39.21	1.05	1.05	-0.11
42M	Testes	49.30	45.41	1.05	1.05	0.13
43	Adrenal glands	12.84	19.49	1.03	1.03	-0.07
44	Oesophagus wall	13.73	19.69	1.03	1.03	-0.06
45	Generic soft tissue	12.24	19.82	1.03	1.03	-0.10
46F	Uterus and cervix	0.86	16.73	1.03	1.03	-0.09
46M	Prostate	12.42	19.94	1.03	1.03	-0.10
47	Lymphatic nodes	10.04	19.25	1.03	1.03	-0.12
48F	Breast glandular tissue	-44.72	2.48	1.04	1.04	0.06
48M	Breast glandular tissue	-50.41	-0.59	1.04	1.04	0.02
49	Adipose tissue	-115.30	-60.81	0.98	0.98	0.12
50	Lung tissue	-563.08	-568.05	0.43	0.43	0.07
51	GI tract contents	21.19	19.16	1.02	1.02	-0.05
52	Urine	16.51	8.51	1.00	1.01	0.38

TABLE XIV: DECT: CT numbers, reference (RSP_{ref}) and predicted RSP (RSP_{DECT}) values, and RSP errors for tissue compositions in a 15-year old female.

ID	Tissue type	CT number [HU] 80 kVp	CT number [HU] 140 kVp (Sn)	RSP_{ref}	RSP_{DECT}	Δ RSP_{DECT} [%]
1	Teeth	5492.10	2774.66	2.57	2.50	-2.68
2	Mineral Bone	2513.33	1189.30	1.61	1.62	0.72
3	Humeri, upper half, spongiosa	305.79	164.49	1.12	1.10	-1.15
4	Humeri, lower half, spongiosa	71.44	127.09	1.17	1.17	0.06
5	Ulnae and radii, spongiosa	72.28	134.45	1.18	1.18	0.10
6	Wrists and hand, spongiosa	-12.11	52.30	1.10	1.10	0.18
7	Clavicles, spongiosa	288.61	169.27	1.13	1.12	-0.76
8	Cranium, spongiosa	663.98	341.78	1.21	1.21	-0.09
9	Femora, upper half, spongiosa	345.37	201.29	1.15	1.14	-0.76
10	Femora, lower half, spongiosa	461.35	257.15	1.18	1.17	-0.82
11	Tibiae, fibulae, patellae, spongiosa	314.28	132.81	1.07	1.06	-0.81
12	Ankles and foot, spongiosa	293.25	122.62	1.06	1.05	-0.89
13	Mandible, spongiosa	584.27	301.69	1.19	1.18	-0.17
14	Pelvis, spongiosa	127.76	94.03	1.09	1.08	-0.98
15	Ribs, spongiosa	206.03	125.04	1.10	1.09	-0.90
16	Scapulae, spongiosa	452.76	284.14	1.22	1.21	-0.57
17	Cervical spine, spongiosa	273.43	163.01	1.12	1.12	-0.78
18	Thoracic spine, spongiosa	464.29	189.50	1.08	1.07	-0.10
19	Lumbar spine, spongiosa	293.68	137.32	1.08	1.07	-0.65
20	Sacrum, spongiosa	161.31	73.41	1.04	1.04	-0.92
21	Sternum, spongiosa	200.49	107.76	1.08	1.07	-0.86
22	Humeri, femora, upper half, medullary cavity	286.19	60.14	0.97	0.97	-0.50
23	Humeri, femora, lower half, medullary cavity	103.18	12.65	0.99	0.97	-1.36
24	Ulnae and radii, medullary cavity	-97.18	-41.94	1.00	1.00	0.13
25	Tibiae, fibulae, patellae, medullary cavity	187.30	24.37	0.96	0.96	-0.85
26	Cartilage	140.33	99.13	1.08	1.08	0.18
27	Skin	77.61	84.84	1.09	1.09	0.09
28	Blood vessels	66.69	54.81	1.05	1.05	0.07
29	Oral mucosa	18.30	12.66	1.01	1.01	0.05
30	Liver	44.95	42.10	1.04	1.04	0.00
31	Pancreas	9.83	12.19	1.02	1.02	0.06
32	Brain	44.09	37.30	1.04	1.04	-0.08
33	Heart wall	37.27	33.49	1.03	1.04	0.05
34	Eye lens and bulb	19.22	30.37	1.04	1.04	0.25
35	Kidney tissue	39.22	33.39	1.03	1.03	0.08
36	Stomach wall	20.61	22.59	1.03	1.03	0.18
37	Small intestine wall	20.48	22.49	1.03	1.03	0.18
38	Colon wall, rectum	20.48	22.49	1.03	1.03	0.18
39	Spleen	60.02	53.59	1.05	1.05	0.11
40	Thyroid	95.52	61.55	1.04	1.05	0.35
41	Urinary bladder wall	46.66	36.85	1.03	1.04	0.07
42	Ovaries	30.10	39.17	1.05	1.05	-0.11
43	Adrenal glands	2.43	9.43	1.02	1.02	-0.07
44	Oesophagus wall	13.13	19.58	1.03	1.03	-0.07
45	Generic soft tissue	0.81	9.52	1.02	1.02	-0.11
46	Uterus and cervix	-1.00	16.76	1.03	1.03	-0.13
47	Lymphatic nodes	0.10	9.31	1.02	1.02	-0.12
48	Breast glandular tissue	-48.65	1.89	1.04	1.04	0.08
49	Adipose tissue	-133.84	-80.47	0.96	0.96	0.12
50	Lung tissue	-692.95	-696.44	0.30	0.30	0.07
51	GI tract contents	11.28	9.27	1.01	1.01	-0.05
52	Urine	16.51	8.51	1.00	1.01	0.38

TABLE XV: DECT: CT numbers, reference (RSP_{ref}) and predicted RSP (RSP_{DECT}) values, and RSP errors for tissue compositions in a 15-year old male.

ID	Tissue type	CT number [HU] 80 kVp	CT number [HU] 140 kVp (Sn)	RSP_{ref}	RSP_{DECT}	Δ RSP_{DECT} [%]
1	Teeth	5492.10	2774.66	2.57	2.50	-2.68
2	Mineral Bone	2513.33	1189.30	1.61	1.62	0.72
3	Humeri, upper half, spongiosa	305.16	164.30	1.12	1.10	-1.16
4	Humeri, lower half, spongiosa	71.47	128.45	1.17	1.17	0.05
5	Ulnae and radii, spongiosa	72.22	135.14	1.18	1.18	0.11
6	Wrists and hand, spongiosa	-11.65	53.15	1.10	1.10	0.18
7	Clavicles, spongiosa	290.96	169.91	1.13	1.12	-0.78
8	Cranium, spongiosa	669.03	340.41	1.20	1.20	-0.10
9	Femora, upper half, spongiosa	349.77	202.54	1.15	1.14	-0.76
10	Femora, lower half, spongiosa	461.09	257.14	1.18	1.17	-0.83
11	Tibiae, fibulae, patellae, spongiosa	318.04	133.78	1.07	1.06	-0.82
12	Ankles and foot, spongiosa	294.12	123.62	1.06	1.05	-0.91
13	Mandible, spongiosa	592.16	303.81	1.19	1.18	-0.19
14	Pelvis, spongiosa	131.96	95.89	1.09	1.08	-1.03
15	Ribs, spongiosa	204.79	125.46	1.10	1.09	-0.93
16	Scapulae, spongiosa	455.46	285.75	1.22	1.21	-0.60
17	Cervical spine, spongiosa	275.78	163.64	1.12	1.12	-0.81
18	Thoracic spine, spongiosa	472.93	192.01	1.08	1.07	-0.13
19	Lumbar spine, spongiosa	297.18	138.27	1.08	1.07	-0.67
20	Sacrum, spongiosa	163.41	74.84	1.05	1.04	-0.95
21	Sternum, spongiosa	202.63	109.20	1.08	1.07	-0.89
22	Humeri, femora, upper half, medullary cavity	289.31	60.37	0.97	0.97	-0.49
23	Humeri, femora, lower half, medullary cavity	102.62	12.48	0.99	0.97	-1.37
24	Ulnae and radii, medullary cavity	-97.68	-42.02	1.00	1.00	0.13
25	Tibiae, fibulae, patellae, medullary cavity	191.10	24.92	0.96	0.96	-0.84
26	Cartilage	138.78	98.79	1.08	1.08	0.18
27	Skin	77.57	84.76	1.09	1.09	0.09
28	Blood vessels	66.69	54.81	1.05	1.05	0.07
29	Oral mucosa	28.28	22.58	1.02	1.02	0.05
30	Liver	45.40	42.16	1.04	1.04	0.01
31	Pancreas	20.22	22.26	1.03	1.03	0.06
32	Brain	44.09	37.23	1.04	1.04	-0.08
33	Heart wall	37.41	33.53	1.03	1.04	0.05
34	Eye lens and bulb	19.22	30.37	1.04	1.04	0.25
35	Kidney tissue	39.51	33.47	1.03	1.03	0.08
36	Stomach wall	20.71	22.69	1.03	1.03	0.17
37	Small intestine wall	20.58	22.58	1.03	1.03	0.17
38	Colon wall, rectum	20.58	22.58	1.03	1.03	0.17
39	Spleen	68.13	55.18	1.05	1.05	0.06
40	Thyroid	95.58	61.56	1.04	1.05	0.35
41	Urinary bladder wall	46.66	36.85	1.03	1.04	0.07
42	Testes	49.30	45.41	1.05	1.05	0.13
43	Adrenal glands	13.71	19.75	1.03	1.03	-0.06
44	Oesophagus wall	14.70	19.90	1.03	1.03	-0.05
45	Generic soft tissue	12.51	19.33	1.03	1.03	-0.07
46	Prostate	12.51	19.33	1.03	1.03	-0.07
47	Lymphatic nodes	10.19	19.29	1.03	1.03	-0.12
48	Breast glandular tissue	-48.35	0.01	1.04	1.04	0.01
49	Adipose tissue	-133.66	-80.42	0.96	0.96	0.12
50	Lung tissue	-638.58	-642.69	0.36	0.36	0.07
51	GI tract contents	21.19	19.16	1.02	1.02	-0.05
52	Urine	16.51	8.51	1.00	1.01	0.38

TABLE XVI: DECT: CT numbers, reference (RSP_{ref}) and predicted RSP (RSP_{DECT}) values, and RSP errors for tissue compositions in the adult female.

ID	Tissue type	CT number [HU] 80 kVp	CT number [HU] 140 kVp (Sn)	RSP_{ref}	RSP_{DECT}	Δ RSP_{DECT} [%]
1	Teeth	5585.89	2651.85	2.36	2.04	-13.37
2	Mineral Bone	2847.68	1359.38	1.70	1.71	0.52
3	Humeri, upper half, spongiosa	517.95	271.26	1.17	1.17	-0.37
4	Humeri, lower half, spongiosa	288.20	145.29	1.10	1.08	-1.06
5	Ulnae and radii, spongiosa	288.20	145.29	1.10	1.08	-1.06
6	Wrists and hand, spongiosa	288.20	145.29	1.10	1.08	-1.06
7	Clavicles, spongiosa	462.36	217.73	1.12	1.12	-0.35
8	Cranium, spongiosa	553.67	242.93	1.11	1.11	-0.09
9	Femora, upper half, spongiosa	158.39	126.50	1.13	1.11	-1.26
10	Femora, lower half, spongiosa	288.20	145.29	1.10	1.08	-1.06
11	Tibiae, fibulae, patellae, spongiosa	288.20	145.29	1.10	1.08	-1.06
12	Ankles and foot, spongiosa	288.20	145.29	1.10	1.08	-1.06
13	Mandible, spongiosa	553.66	297.42	1.19	1.19	-0.34
14	Pelvis, spongiosa	273.61	152.15	1.11	1.10	-0.93
15	Ribs, spongiosa	281.97	185.19	1.15	1.14	-0.92
16	Scapulae, spongiosa	383.35	223.70	1.17	1.16	-0.82
17	Cervical spine, spongiosa	226.37	84.51	1.03	1.02	-0.61
18	Thoracic spine, spongiosa	170.73	90.21	1.07	1.06	-0.96
19	Lumbar spine, spongiosa	361.30	163.07	1.08	1.08	-0.33
20	Sacrum, spongiosa	59.92	31.47	1.03	1.02	-0.98
21	Sternum, spongiosa	119.38	53.10	1.03	1.02	-1.00
22	Humeri, femora, upper half, medullary cavity	-102.17	-42.27	1.00	1.00	0.17
23	Humeri, femora, lower half, medullary cavity	-102.17	-42.27	1.00	1.00	0.17
24	Ulnae and radii, medullary cavity	-102.17	-42.27	1.00	1.00	0.17
25	Tibiae, fibulae, patellae, medullary cavity	-102.17	-42.27	1.00	1.00	0.17
26	Cartilage	140.33	99.13	1.08	1.08	0.18
27	Skin	67.96	75.02	1.08	1.08	0.09
28	Blood vessels	66.69	54.81	1.05	1.05	0.07
29	Oral mucosa	48.25	42.44	1.04	1.04	0.05
30	Liver	49.30	42.72	1.04	1.04	0.08
31	Pancreas	39.68	42.01	1.05	1.05	0.06
32	Brain	54.13	47.27	1.05	1.05	-0.08
33	Heart wall	46.94	43.40	1.04	1.05	0.04
34	Eye lens and bulb	21.74	31.76	1.04	1.04	0.25
35	Kidney tissue	49.42	43.45	1.04	1.04	0.08
36	Stomach wall	30.38	32.41	1.04	1.04	0.18
37	Small intestine wall	30.38	32.41	1.04	1.04	0.18
38	Colon wall, rectum	30.38	32.41	1.04	1.04	0.18
39	Spleen	40.02	33.71	1.03	1.03	0.11
40	Thyroid	85.09	51.44	1.03	1.04	0.35
41	Urinary bladder wall	46.66	36.85	1.03	1.04	0.07
42	Ovaries	40.96	35.32	1.03	1.04	0.14
43	Adrenal glands	12.51	19.33	1.03	1.03	-0.07
44	Oesophagus wall	13.38	19.58	1.03	1.03	-0.06
45	Generic soft tissue	11.98	19.82	1.03	1.03	-0.10
46	Uterus and cervix	0.40	16.53	1.03	1.03	-0.09
47	Lymphatic nodes	33.94	28.26	1.03	1.03	0.25
48	Breast glandular tissue	-45.43	2.20	1.04	1.04	0.07
49	Adipose tissue	-123.70	-70.44	0.97	0.97	0.09
50	Lung tissue	-617.27	-620.12	0.38	0.38	0.11
51	GI tract contents	31.11	29.06	1.03	1.03	-0.05
52	Urine	46.70	38.47	1.03	1.04	0.38

TABLE XVII: DECT: CT numbers, reference (RSP_{ref}) and predicted RSP (RSP_{DECT}) values, and RSP errors for tissue compositions in the adult male.

ID	Tissue type	CT number [HU] 80 kVp	CT number [HU] 140 kVp (Sn)	RSP_{ref}	RSP_{DECT}	Δ RSP_{DECT} [%]
1	Teeth	5585.89	2651.85	2.36	2.04	-13.37
2	Mineral Bone	2847.68	1359.38	1.70	1.71	0.52
3	Humeri, upper half, spongiosa	531.16	273.51	1.17	1.17	-0.05
4	Humeri, lower half, spongiosa	261.20	138.58	1.10	1.09	-1.02
5	Ulnae and radii, spongiosa	261.20	138.58	1.10	1.09	-1.02
6	Wrists and hand, spongiosa	261.20	138.58	1.10	1.09	-1.02
7	Clavicles, spongiosa	374.14	196.29	1.13	1.12	-0.50
8	Cranium, spongiosa	394.51	205.44	1.13	1.13	-0.42
9	Femora, upper half, spongiosa	297.25	157.95	1.11	1.10	-0.76
10	Femora, lower half, spongiosa	261.20	138.58	1.10	1.09	-1.02
11	Tibiae, fibulae, patellae, spongiosa	261.20	138.58	1.10	1.09	-1.02
12	Ankles and foot, spongiosa	261.20	138.58	1.10	1.09	-1.02
13	Mandible, spongiosa	600.60	307.39	1.18	1.19	0.07
14	Pelvis, spongiosa	290.14	155.29	1.11	1.10	-0.70
15	Ribs, spongiosa	407.57	214.09	1.14	1.13	-0.26
16	Scapulae, spongiosa	470.07	242.56	1.15	1.15	-0.19
17	Cervical spine, spongiosa	72.39	49.20	1.05	1.04	-0.93
18	Thoracic spine, spongiosa	143.51	83.37	1.07	1.06	-0.92
19	Lumbar spine, spongiosa	246.77	136.27	1.10	1.09	-0.71
20	Sacrum, spongiosa	19.95	22.35	1.04	1.03	-0.75
21	Sternum, spongiosa	47.37	36.46	1.04	1.03	-0.86
22	Humeri, femora, upper half, medullary cavity	-102.03	-42.23	1.00	1.00	0.17
23	Humeri, femora, lower half, medullary cavity	-102.03	-42.23	1.00	1.00	0.17
24	Ulnae and radii, medullary cavity	-102.03	-42.23	1.00	1.00	0.17
25	Tibiae, fibulae, patellae, medullary cavity	-102.03	-42.23	1.00	1.00	0.17
26	Cartilage	140.33	99.13	1.08	1.08	0.18
27	Skin	67.96	75.02	1.08	1.08	0.09
28	Blood	66.69	54.81	1.05	1.05	0.07
29	MuscleTissue	48.25	42.44	1.04	1.04	0.05
30	Liver	49.44	42.76	1.04	1.04	0.09
31	Pancreas	39.89	42.07	1.05	1.05	0.06
32	Brain	54.19	47.29	1.05	1.05	-0.08
33	Heart	56.91	53.34	1.05	1.06	0.04
34	Eyes	22.12	31.87	1.04	1.04	0.25
35	Kidneys	49.56	43.49	1.04	1.04	0.08
36	Stomach	30.38	32.41	1.04	1.04	0.18
37	SmallIntestine	30.44	32.43	1.04	1.04	0.18
38	LargeIntestine	30.44	32.43	1.04	1.04	0.18
39	Spleen	47.97	35.27	1.03	1.03	0.06
40	Thyroid	85.15	51.45	1.03	1.04	0.35
41	UrinaryBladder	46.66	36.85	1.03	1.04	0.07
42	Testes	39.36	35.54	1.04	1.04	0.12
43	Adrenals	13.53	19.63	1.03	1.03	-0.06
44	Oesophagus	14.60	19.94	1.03	1.03	-0.05
45	Generic soft tissue	12.08	19.20	1.03	1.03	-0.07
46	Prostate	12.08	19.20	1.03	1.03	-0.07
47	Lymph	33.94	28.26	1.03	1.03	0.25
48	Breast	-49.97	-0.53	1.04	1.04	0.02
49	Adipose tissue	-123.64	-70.42	0.97	0.97	0.09
50	LungTissueCompressed	-617.27	-620.12	0.38	0.38	0.11
51	GI tract contents	31.11	29.06	1.03	1.03	-0.05
52	Urine	46.70	38.47	1.03	1.04	0.38

Appendix E: Statistics of the RSP errors for SECT calibration curves derived with different simulated X-ray spectra

TABLE XVIII: Percentage mean RSP errors, standard deviations and minimum and maximum errors for all age groups (female and male) estimated with SECT using an 80 kVp simulated spectrum (upper part of the table), a 100 kVp simulated spectrum (middle part), and a 120 kVp simulated spectrum (lower part). Teeth and air inside the body are excluded from the statistics.

Age groups	Soft tissues				Bones			
**SECT 80 kVp**	μ	σ	min	max	μ	σ	min	max
Newborn female	2.29	4.55	-3.19	15.57	-3.95	5.25	-15.69	2.26
1-year old female	2.13	5.19	-3.53	19.13	-3.53	6.91	-20.77	5.30
5-year old female	1.66	3.80	-3.52	15.03	-3.18	5.39	-15.21	3.62
10-year old female	1.60	3.49	-3.53	11.54	-1.98	4.72	-12.95	4.90
15-year old female	1.62	3.54	-3.53	13.79	-1.47	4.52	-10.90	6.35
Adult female	0.27	1.42	-2.90	5.91	0.40	2.82	-4.65	5.99
Newborn male	2.30	4.55	-3.19	15.57	-3.95	5.25	-15.69	2.26
1-year old male	2.17	5.17	-3.53	19.13	-3.53	6.91	-20.77	5.30
5-year old male	1.70	3.78	-3.52	15.03	-3.18	5.39	-15.21	3.62
10-year old male	1.64	3.47	-3.53	11.54	-1.98	4.72	-12.95	4.90
15-year old male	1.66	3.61	-3.52	14.00	-1.47	4.57	-10.99	6.51
Adult male	0.29	1.44	-2.90	5.91	-0.11	0.62	-2.37	0.36
**SECT 100 kVp**	μ	σ	min	max	μ	σ	min	max
Newborn female	2.15	4.34	-2.99	14.97	-3.68	5.00	-14.84	2.25
1-year old female	2.00	4.97	-3.32	18.36	-3.29	6.58	-19.70	5.15
5-year old female	1.54	3.62	-3.31	14.43	-2.94	5.13	-14.40	3.54
10-year old female	1.47	3.25	-3.32	11.00	-1.81	4.50	-12.22	4.79
15-year old female	1.50	3.35	-3.32	13.25	-1.35	4.31	-10.32	6.15
Adult female	0.24	1.27	-2.74	5.06	0.43	2.68	-4.44	5.68
Newborn male	2.15	4.34	-2.99	14.97	-3.68	5.00	-14.84	2.25
1-year old male	2.04	4.95	-3.32	18.36	-3.29	6.58	-19.70	5.15
5-year old male	1.58	3.60	-3.31	14.43	-2.94	5.13	-14.40	3.54
10-year old male	1.51	3.23	-3.32	11.00	-1.81	4.50	-12.22	4.79
15-year old male	1.54	3.40	-3.31	13.45	-1.35	4.35	-10.41	6.30
Adult male	0.26	1.29	-2.74	5.06	-0.05	0.61	-2.32	0.36
**SECT 120 kVp**	μ	σ	min	max	μ	σ	min	max
Newborn female	2.04	4.20	-2.84	14.57	-3.46	4.81	-14.20	2.25
1-year old female	1.90	4.83	-3.18	17.83	-3.09	6.34	-18.90	5.07
5-year old female	1.45	3.48	-3.17	14.03	-2.75	4.93	-13.77	3.51
10-year old female	1.35	3.07	-3.17	10.46	-1.67	4.34	-11.67	4.74
15-year old female	1.40	3.19	-3.18	12.71	-1.24	4.17	-9.89	6.04
Adult female	0.23	1.16	-2.62	4.48	0.47	2.57	-4.26	5.41
Newborn male	2.04	4.20	-2.84	14.57	-3.46	4.81	-14.20	2.25
1-year old male	1.94	4.81	-3.18	17.83	-3.09	6.34	-18.90	5.07
5-year old male	1.48	3.46	-3.17	14.03	-2.75	4.93	-13.77	3.51
10-year old male	1.39	3.06	-3.17	10.46	-1.67	4.34	-11.67	4.74
15-year old male	1.43	3.24	-3.17	12.91	-1.24	4.21	-9.98	6.18
Adult male	0.24	1.18	-2.62	4.48	0.02	0.63	-2.30	0.46

Appendix F: Positions of paediatric tissues relative to the calibration curve

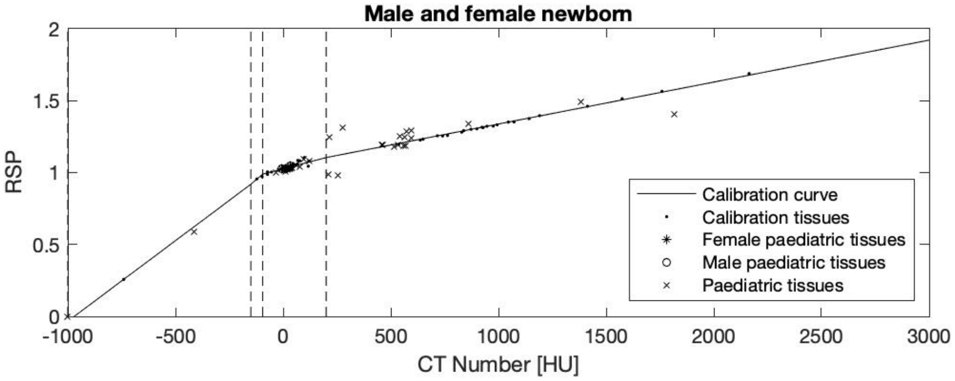

(a)

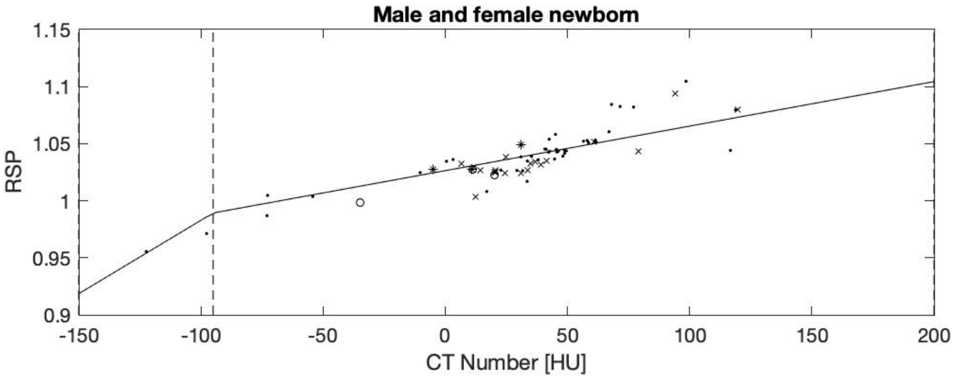

(b)

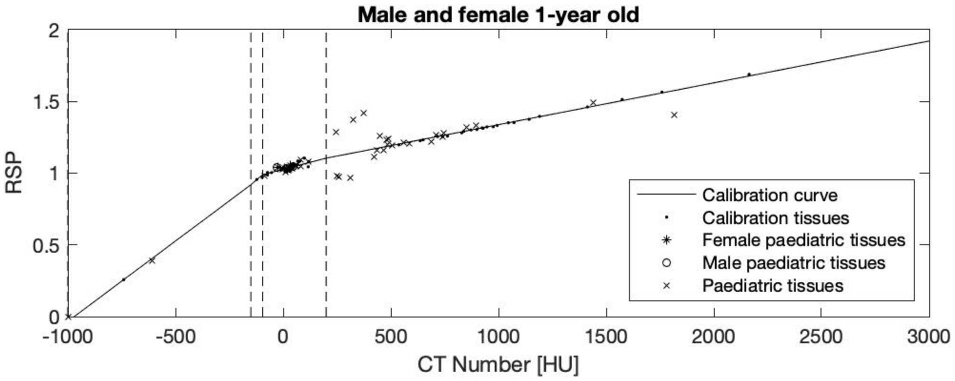

(c)

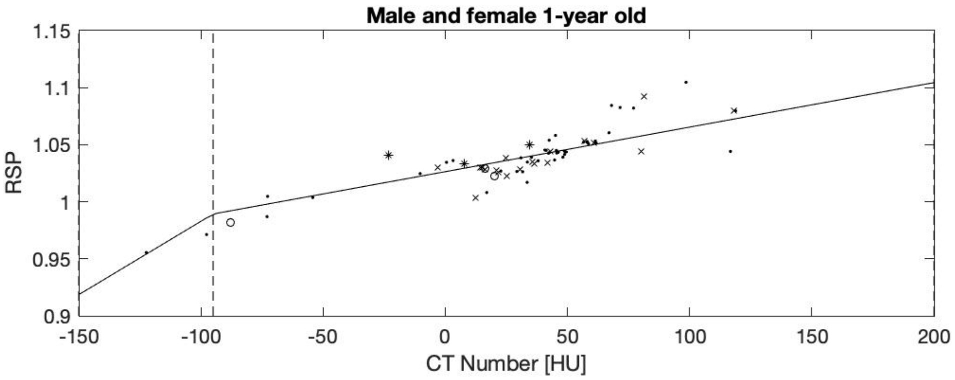

(d)

FIG. 2: Positions of the paediatric tissues relative to the stoichiometric calibration curve and zoom into the soft tissue region. The dots represent the reference tissues (adult), the line is the piece-wise fit (Schneider *et al.* 1996) and the crosses represent the paediatric tissues (except teeth) of a newborn (a and b) and a 1-year old child (c and d). Tissues that differ in male and female children are marked with a circle (male) or an asterix (female).

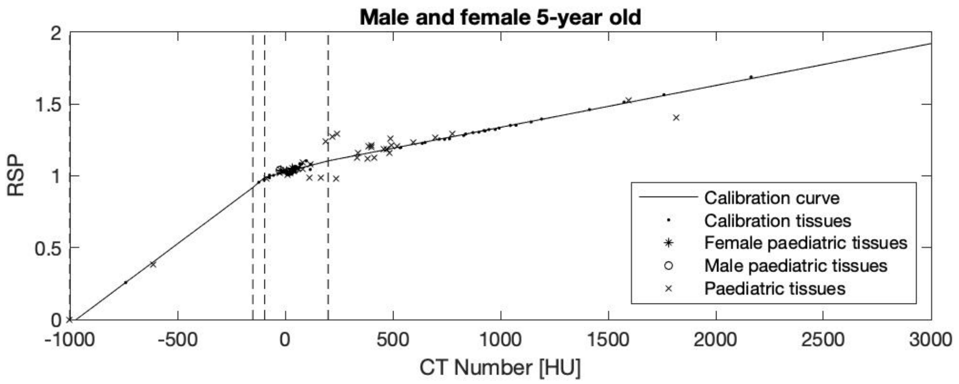

(a)

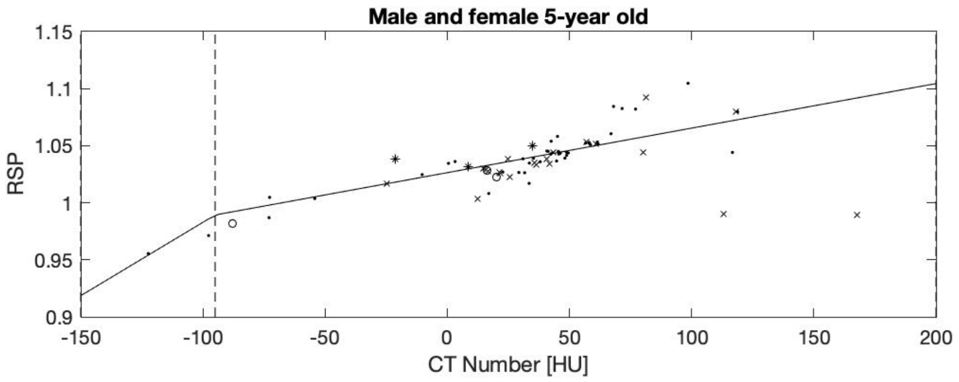

(b)

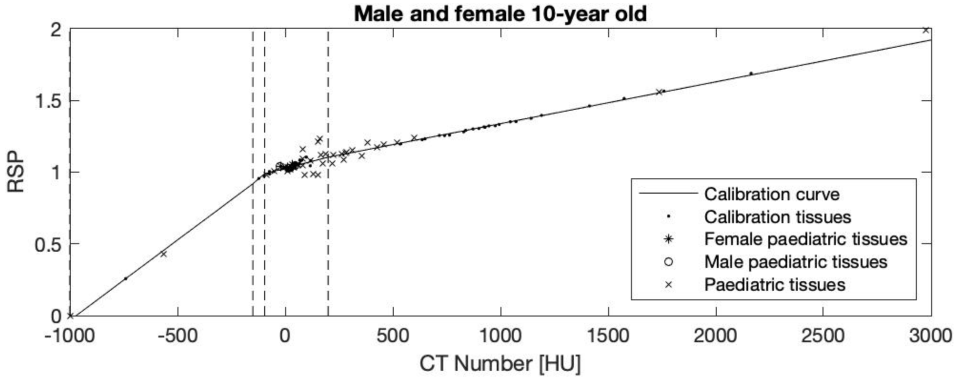

(c)

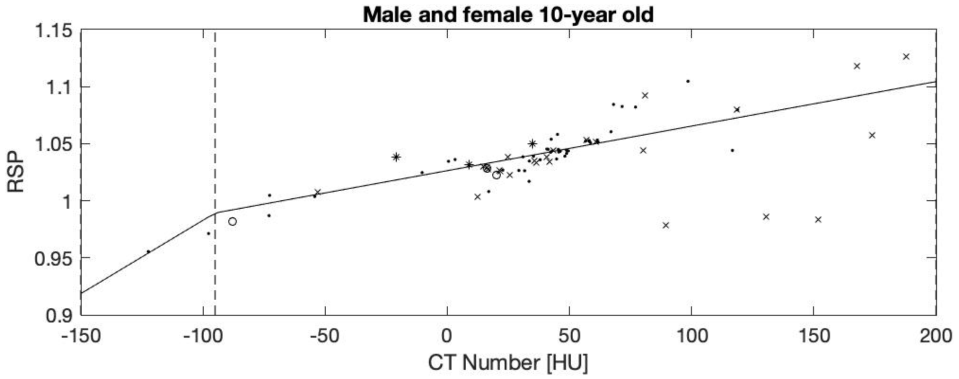

(d)

FIG. 3: Positions of the paediatric tissues relative to the stoichiometric calibration curve and zoom into the soft tissue region. The dots represent the reference tissues (adult), the line is the piece-wise fit (Schneider *et al.* 1996) and the crosses represent the paediatric tissues (except teeth) of a 5-year old child (a and b) and a 10-year old child (c and d). Tissues that differ in male and female children are marked with a circle (male) or an asterix (female).

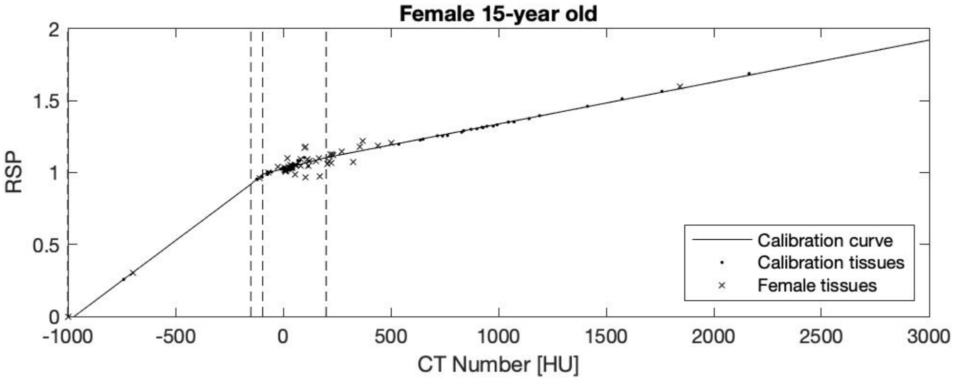

(a)

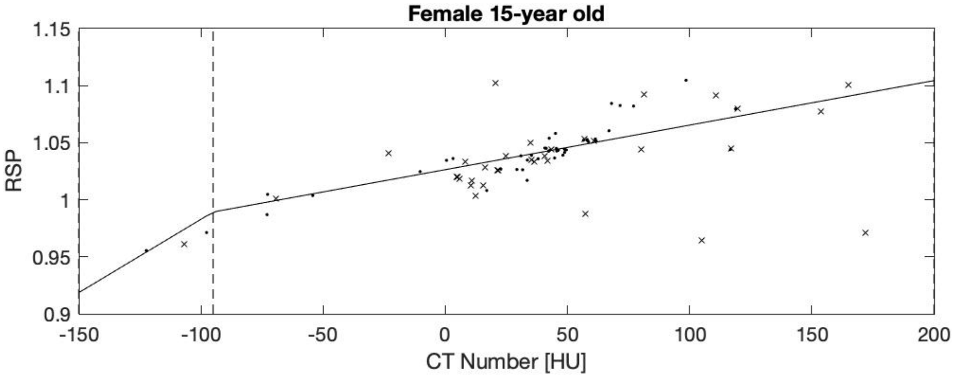

(b)

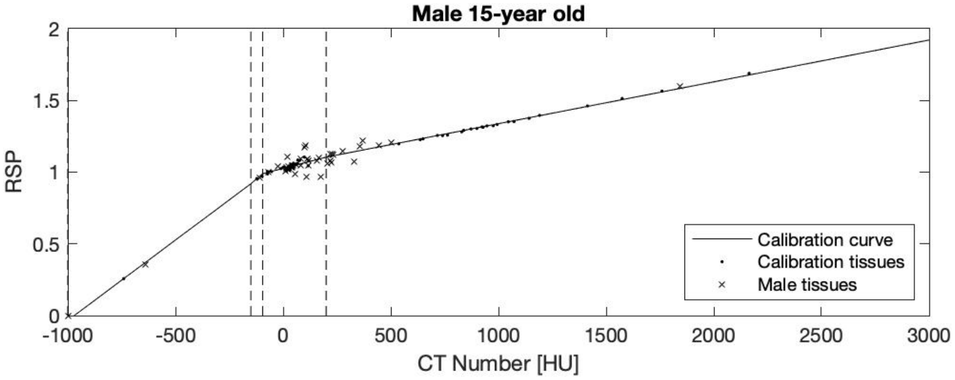

(c)

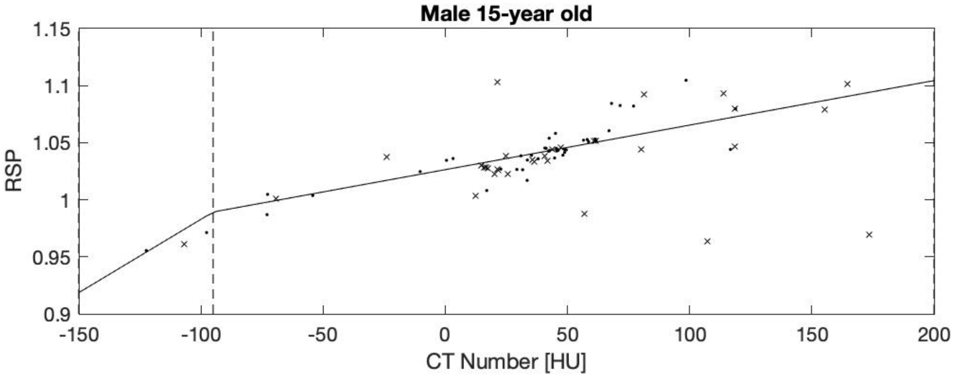

(d)

FIG. 4: Positions of the paediatric tissues relative to the stoichiometric calibration curve and zoom into the soft tissue region. The dots represent the reference tissues (adult), the line is the piece-wise fit (Schneider *et al.* 1996) and the crosses represent the paediatric tissues (except teeth) of a 15-year old female (a and b) and a 15-year old male (c and d).

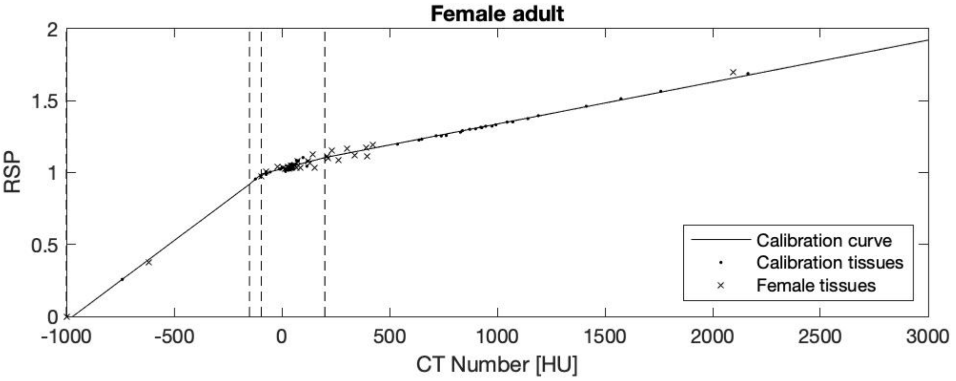

(a)

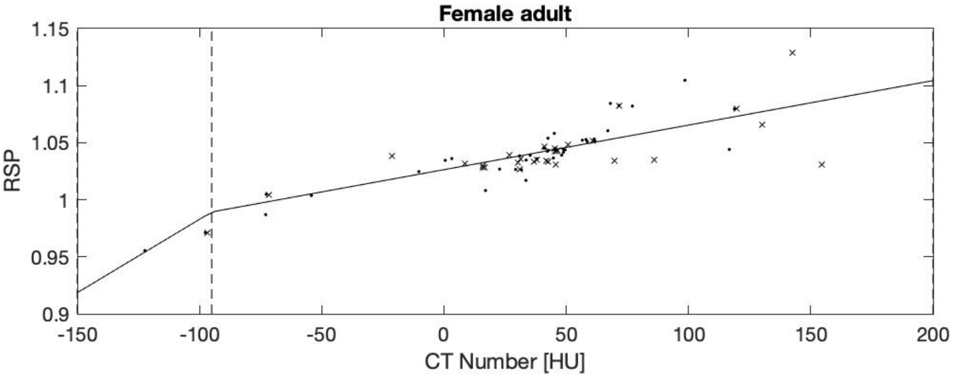

(b)

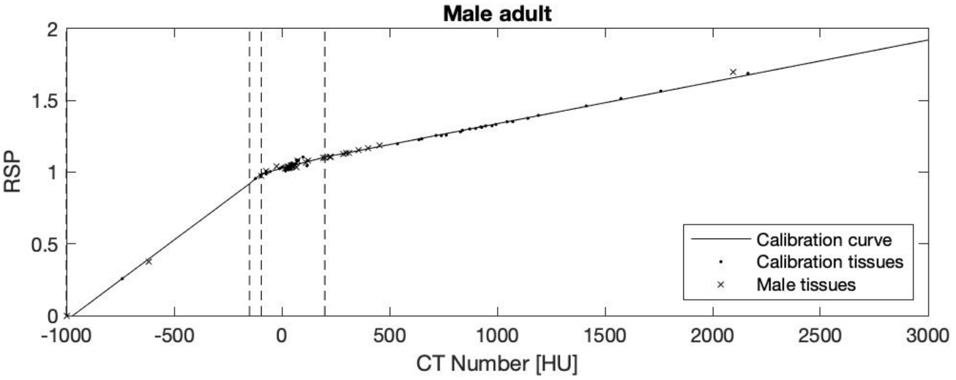

(c)

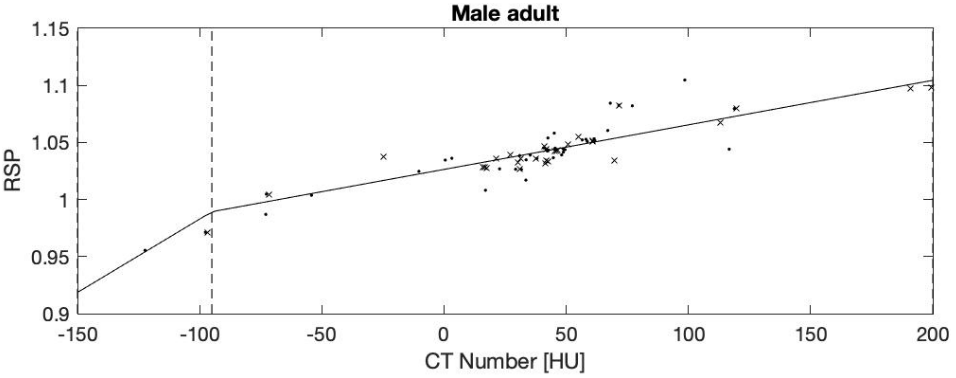

(d)

FIG. 5: Positions of the adult tissues relative to the stoichiometric calibration curve and zoom into the soft tissue region. The dots represent the reference tissues (adult, Woodard and White), the line is the piece-wise fit (Schneider *et al.* 1996) and the crosses represent the testing tissues of an adult male (ICRP publication 143) (a and b) and an adult female (c and d).

Appendix G: DHV Analysis

TABLE XIX: DVH metrics of the target volumes

		D_{min} [Gy]	D_{max} [Gy]	D_{mean} [Gy]	D_{95} [Gy]
Ewing's phantom low dose CTV	RSP_{SECT}	43.42	52.06	47.34	44.73
	RSP_{DECT}	43.37	52.08	47.37	44.69
	RSP_{ref}	43.40	52.08	47.36	44.69
Ewing's phantom high dose CTV	RSP_{SECT}	48.43	52.06	50.43	49.63
	RSP_{DECT}	48.30	52.08	50.41	49.60
	RSP_{ref}	48.30	52.08	50.41	49.60
Salivary phantom CTV	RSP_{SECT}	58.77	68.07	64.95	63.97
	RSP_{DECT}	58.92	68.16	64.95	63.98
	RSP_{ref}	58.92	68.14	64.95	63.97
Glioma phantom CTV	RSP_{SECT}	49.41	56.97	54.00	53.07
	RSP_{DECT}	49.25	57.02	54.00	53.07
	RSP_{ref}	49.30	57.02	54.00	53.07

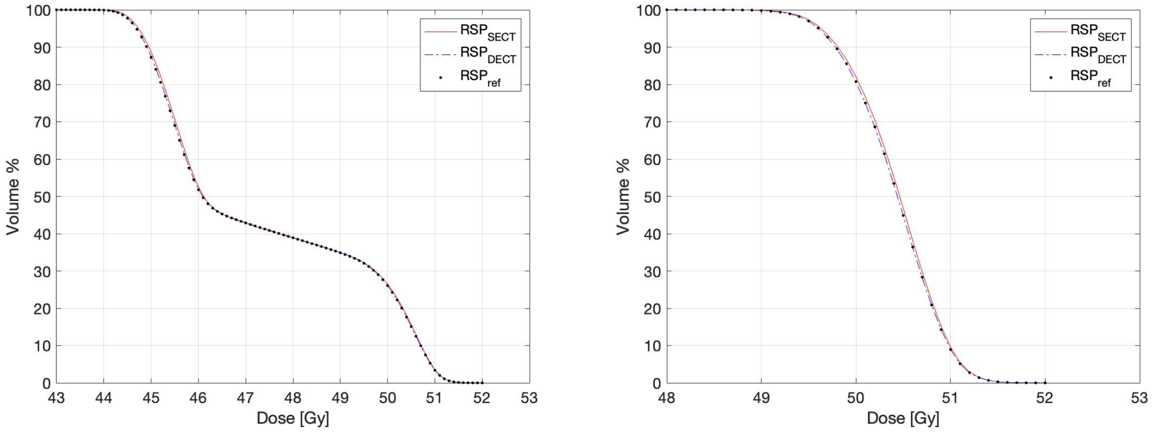

(a) (b)

FIG. 6: Example DVHs of the target volumes for the Ewing's sarcoma. (a) shows the CTV DVH of the low-dose CTV, (b) the CTV DVH of the high dose CTV. Note that both graphs are zoomed in on the x-axis, only displaying the steep fall-off in the DVH.
